# Allele-Specific Silencing of Mutant Huntingtin in Rodent Brain and Human Stem Cells

**DOI:** 10.1371/journal.pone.0099341

**Published:** 2014-06-13

**Authors:** Valérie Drouet, Marta Ruiz, Diana Zala, Maxime Feyeux, Gwennaëlle Auregan, Karine Cambon, Laetitia Troquier, Johann Carpentier, Sophie Aubert, Nicolas Merienne, Fany Bourgois-Rocha, Raymonde Hassig, Maria Rey, Noëlle Dufour, Frédéric Saudou, Anselme L. Perrier, Philippe Hantraye, Nicole Déglon

**Affiliations:** 1 Institute of Biomedical Imaging (I2BM) and Molecular Imaging Research Center (MIRCen), Atomic Energy Commission (CEA), Fontenay-aux-Roses, France; 2 URA2210, Centre National de Recherché Scientifique (CNRS), Fontenay-aux-Roses, France; 3 Institut Curie, Orsay, France; 4 UMR3306, Centre National de Recherché Scientifique (CNRS), Orsay, France; 5 U1005, Institut National de la Santé et de la Recherche Médicale (INSERM), Orsay France; 6 U861, Institut National de la Santé et de la Recherche Médicale (INSERM), AFM, Evry, France; 7 UEVE U861, I-STEM, AFM, Evry, France; 8 CECS, I-STEM, AFM, Evry, France; 9 Department of Clinical Neurosciences (DNC), Lausanne University Hospital (CHUV), Lausanne, Switzerland; Inserm U837, France

## Abstract

Huntington's disease (HD) is an autosomal dominant neurodegenerative disorder resulting from polyglutamine expansion in the huntingtin (HTT) protein and for which there is no cure. Although suppression of both wild type and mutant *HTT* expression by RNA interference is a promising therapeutic strategy, a selective silencing of mutant *HTT* represents the safest approach preserving WT *HTT* expression and functions. We developed small hairpin RNAs (shRNAs) targeting single nucleotide polymorphisms (SNP) present in the *HTT* gene to selectively target the disease *HTT* isoform. Most of these shRNAs silenced, efficiently and selectively, mutant *HTT in vitro*. Lentiviral-mediated infection with the shRNAs led to selective degradation of mutant *HTT* mRNA and prevented the apparition of neuropathology in HD rat's striatum expressing mutant HTT containing the various SNPs. In transgenic BACHD mice, the mutant *HTT* allele was also silenced by this approach, further demonstrating the potential for allele-specific silencing. Finally, the allele-specific silencing of mutant *HTT* in human embryonic stem cells was accompanied by functional recovery of the vesicular transport of BDNF along microtubules. These findings provide evidence of the therapeutic potential of allele-specific RNA interference for HD.

## Introduction

HD is an autosomal dominant neurodegenerative disease that affects 1 in 10,000 adults [1]. The symptoms, which include progressive motor, psychiatric and cognitive dysfunctions, are associated with the degeneration of the major population of striatal neurons, the GABAergic spiny projection neurons. The mutation underlying HD is an expansion of a trinucleotide CAG repeat which encodes a polyglutamine (polyQ) tract in the N-terminal region of the HTT protein [2]. This mutation confers a new toxic function on the protein, in part through the production of short N-terminal fragments carrying the polyglutamine and the accumulation of misfolded HTT [3].

Gene silencing techniques, aiming to reduce intracellular levels of polyglutamine-encoding mRNA, have the potential to halt, or at least delay, the process of neuronal death at its source and are therefore promising for the treatment of polyglutamine (polyQ) diseases [4]. However, most *in vivo* studies have been performed with small interfering RNAs (siRNAs) that do not discriminate between the WT and mutant alleles. Several groups including ours demonstrated that non allele-specific silencing of *HTT with* shRNA is well tolerated up to 9 months in a lentiviral-based HD model [5] and in transgenic N171-82Q mice [6] but leads to transcriptomic changes; the functional consequences of these changes are currently unknown. Thus, an allele-specific silencing of mutant *HTT* is potentially the optimal solution for blocking polyQ pathogenesis.

Recently, strategies based on chemically modified single-stranded RNAs targeting the CAG expansion were developed to selectively target mutant *HTT* [7]–[9]. As an alternative strategy, single nucleotide polymorphisms (SNP) have been used to discriminate between WT and mutant transcripts associated with polyglutamine disorders [10]–[12]. Studies with HD patients indicate that the level of heterozygosity of a limited number of SNP is sufficient to select most HD patients, confirming the feasibility of the approach [13]–[15]. Two studies showed that targeting three SNP would be sufficient to treat 75% of their respective HD cohort [13]. Note that a large proportion of patients of European origin clusters in a single haplotype with a specific set of SNPs [16]–[18].

One challenge is that available HD animal models are not entirely appropriate for assessing the therapeutic efficacy and selectivity of shRNAs targeting SNPs. Knock-in mice do not express the human *HTT* gene [19] and transgenic models expressing short N-terminal fragments of human HTT (R6/2, N171-82Q) or viral-based HD models do not contain the corresponding SNPs [20], [21]. Transgenic mice expressing full-length *HTT* possess only one human allele [22], [23], allowing analysis of efficacy but not selectivity, which is an important aspect of this strategy. Only lately, one fully humanized transgenic mouse model of HD was created, containing two human *HTT* alleles, but it was not available at the time we started the study [24]. Therefore, we developed new HD models based on lentiviral expression of a chimeric mutant *HTT* reporter system. Using these new tools and neural derivatives of HD human embryonic stem cells (hESCs), we tested the efficacy and selectivity of shRNAs targeting SNPs in exons 39, 50, 60 and 67 of the human *HTT* gene and functional recovery associated with this silencing.

## Materials and Methods

### Plasmids and lentiviral vector production

We selected four SNPs within the *HTT* gene: rs363125 (exon 39, A or C), rs362331 (exon 50, C or T), rs2276881 (exon 60, A or G) and rs362307 (exon 67 C or T). We designed eight small-hairpin RNA (shRNA) targeting these *HTT* isoforms and cloned them in lentiviral vectors (Materials and Methods S1, Table S1). To assess the efficacy and the selectivity of these shRNAs, we produced constructs encoding chimeric mutant *HTT*, consisting of the sequence of the first 171 amino acids of the human *HTT* with 82 CAG repeats and fused to a part of *HTT* exons containing the SNP (SIN-W-PGK-htt171-82Q-exon; Materials and Methods S1, Table S2). Each chimeric mutant *HTT* construct contains one SNP of interest.

Lentiviral vectors encoding the various shRNAs and the eight chimeric HTT were produced in 293T cells using a 4-plasmid system as previously described [25]. The particle content of viral batches was determined by p24 antigen ELISA (RETROtek, Gentaur, France). The stocks were stored at −80°C until use.

### *In vivo* experimental design and animals

Two sets of *in vivo* experiments were carried out. First, we validated the chimeric targets and tested shRNAs in 200 g adult male Wistar rats (Iffa Credo/Charles River, Les Oncins, France). Second, BACHD mice expressing full-length mutant HTT with 97Q [23] were used to validate the shRNA therapeutic strategy. BACHD mice were genotyped to identify *HTT* isoform present at SNPs studied. Stereotaxic injections of the lentiviral vectors are described in the Materials and Methods S1.

The animals were housed in a temperature-controlled room and maintained on a 12 h day/night cycle. Food and water were available *ad libitum*. All experiments were carried out in accordance with the European Community directive (86/609/EEC) for the care and use of laboratory animals as well as the Swiss animal welfare laws under the authorization n° VD 2486 and 2487 from the Service de la consommation et des affaires vétérinaires du Canton de Vaud, Switzerland.

### Laser Capture Microdissection and punches

Four weeks after co-infection of chimeric *HTT* construct with fully matched or mismatched shRNA, or shCtrl, the animals were sacrificed by administration of an overdose of sodium pentobarbital. Processing of the brains for laser capture microdissection and RT-qPCR were conducted as previously described with human specific primers [5] (platform profileXpert: www.profilexpert.fr). BACHD mice injected with matched shRNA and shCtrl were sacrificed. One millimeter-thick fresh brain slices were obtained using a mouse brain matrix and punches were sampled from the GFP-positive area under a fluorescent microscope and were quickly lyzed in Trizol and stored at −80°C until RNA extraction.

### Quantitative real-time PCR from 293T cells or BACHD striata

Total RNA was extracted with Trizol reagent 48 hrs post-transfection of 293T cells or 4 weeks post-injection of BACHD mice. RT-qPCR was performed in triplicate with random-primed (Invitrogen, Cergy Pontoise, France) cDNAs generated from 0.3-1 µg total RNA. Quantitative PCR was carried out in a 20 µl reaction volume containing Platinum SYBR Green qPCR super Mix-UDG (Invitrogen Cergy Pontoise, France), and 10 µM of both forward and reverse primers recognizing a sequence of human *HTT* present in the first part of all chimeric constructs and in the BACHD human allele (sequences in Table S3) using a Realplex thermal cycler (Eppendorf). Three to 5 samples from cells and 4–10 samples from the striatum punches were subjected to RT-qPCR analyses. Values for *HTT* mRNA were normalized to a reference: β-actin (*ACTB*) or peptidyl propyl isomerase A (*PPIA*) and are expressed as mean percentages ± SEM relative to the control condition.

### Histological processing

Four to 8 weeks post-lentiviral injection, the animals were given an overdose of sodium pentobarbital and were transcardially perfused with a phosphate solution followed by 4% paraformaldehyde (PFA, Sigma-Aldrich, St. Louis, Missouri, United States) and 10% picric acid for fixation. The brains were processed as previously described [5] for immunohistochemistry for dopamine and cAMP-regulated phosphoprotein of a molecular mass of 32 kDa (DARPP-32), ubiquitin (Ubi) and mutant HTT (EM-48). Pictures were taken using 4x, 20x or 63x objectives on an Olympus AX70 microscope.

### Quantification of DARPP-32 lesions and formation of inclusions

The loss of DARPP-32 expression was analyzed by collecting digitized images of twelve sections per animal (separated by 300 µm) with an optic bench and by quantifying the lesion areas in mm^2^ with image analysis software (MCID Core 7.0, InterFocus Imaging, GE Healthcare Niagara Inc.). Lesion areas in each section were determined as regions poor in DARPP-32 staining relative to the surrounding tissue. The lesion volume for each animal is expressed in mm^3^, calculated as the sum of the total lesion area in mm^2^ of all sections multiplied by the inter-section distance (300 µm). For estimation of the number of ubiquitin-positive HTT inclusions, 12 serial coronal sections of the striatum (separated by 300 µm) were scanned with a 10x objective using a Zeiss Axioplan2 imaging microscope equipped with an automated motorized stage and acquisition system (Mercator Pro V6.50, ExploraNova). All ubiquitin-positive objects with an apparent cross-sectional area comprised between 1 and 50 µm^2^ were measured as previously reported [5]. The same parameters were applied for EM-48 aggregates quantification. Bravais-Pearson correlation was performed to validate the use of Ubiquitin staining as a measure of HTT aggregation (Statistica, Statsoft, Maisons-Alfort, France).

### Neural Stem Cells culture and nucleofection

Neural stem cells (NSCs) were obtained and maintained as described previously [26]. NSC lines were differentiated from the following human ES lines: SA-01 (WT XY, passages 30-83, Cellartis AB, Sweden), H9 (WT XX, passages 40–60, WiCell Research Institute), VUB05 (HD XY, 44 CAG, passages 35–130, AZ-VUB, Belgium [27]), SIVF017 (HD, XY, 40 CAG, passages18–35, Sydney IVF Stem Cells, Australia [28]), SIVF018 (HD XX, 46 CAG, passage 18–30, Sydney IVF Stem Cells, Australia [28]) and Huez2.3 (HD XX, 44 CAG, passages 25–47, IGBMC, Strasbourg [29]).

Genotypes for SNP rs362331 of Huez2.3, VUB05 and SIVF018 NSC lines were analyzed by sequencing the PCR product encompassing this SNP generated using the following primers: 5′-CCCAAACGAAGGTACACGA and 5′-CCTGTTGGCCATCTCTCACC.

For videomicroscopy analysis, NSCs were electroporated with 1 µg BDNF-mCherry, a gift from G. Banker (Oregon Health and Science University) and 4 µg SIN-CWP-GFP-TRE-H1-shCtrl (controls: shLUC or shUNIV), -sh50T or -sh50C (see Table S2). NSCs were plated on glass coverslips previously coated with Poly-L-orninthine and Laminin (Sigma, St. Louis, Missouri, United States). Live videomicroscopy was carried out seven to ten days after transfection using an imaging system as previously described [30] with little modifications (Materials and Methods S1). After videomicroscopy experiments, the neuronal cultures were processed for immunofluorescence for anti-HTT-2166 (Materials and Methods S1).

## Results

### Development of SNP-specific shRNA for the treatment of HD

**Figure 1:**
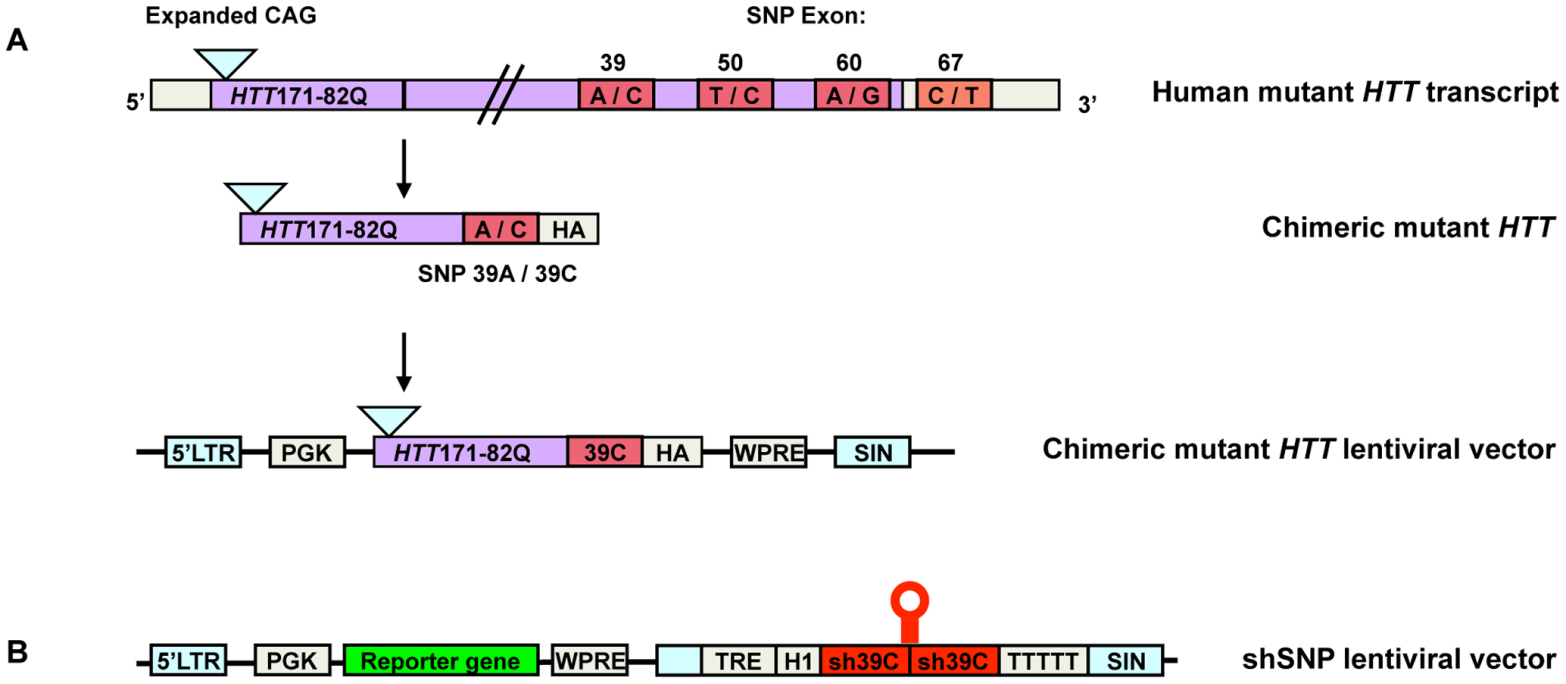
Schematic representation of the chimeric *HTT*- and shSNP-containing lentiviral vectors. (**A**) We chose four SNP within the human *HTT* transcript: exon 39 (rs363125), exon 50 (rs362331), exon 60 (rs2276881) and exon 67 (rs362307). This last SNP is located after the stop codon, in the 3′UTR of the *HTT* gene. The chimeric *HTT* with the exon 39 SNP is illustrated as an example: the sequence surrounding the SNP (A or C) is fused in frame to the 5′ sequence of mutant *HTT* encoding the first 171 amino acids with 82Q. A sequence encoding the HA tag is added at 3′ the end of all fusion proteins to facilitate detection. The fusion construct is then inserted into a SIN transfer vector. (**B**) Representation of a lentiviral vector expressing the shSNP (example of sh39C). The sequence corresponding to the shRNA is inserted downstream from a tetracycline responsive element (TRE) and a H1 promoter in the 3′LTR of the vector. A second expression cassette contains a GFP reporter gene under the control of a PGK promoter. The SNP was located at position 10 or 16 for this particular shSNP, counting from the 5′ end of the guide strand of the shRNA.

To distinguish the normal and disease *HTT* alleles in HD patients, we developed shRNAs targeting the disease isoform of heterozygous single-nucleotide polymorphisms (SNP). We produced eight shRNAs corresponding to four SNP present within the *HTT* gene: in exons 39, 50, 60 and 67 (**Fig. 1A**). The selection was based on their presence in *HTT* exons and a high frequency of heterozygosity in the human population (http://www.ncbi.nlm.nih.gov/snp and [13], [14], [16]). DNAs encoding the shRNAs targeting each SNP (shSNPs: sh39A, sh39C, sh50C, sh50T, sh60A, sh60G and sh67C, sh67T) were inserted into a lentiviral vector [5] (**Fig. 1B**). The SNP was localized at 10 (p10, exons 39, 60 and 67) or 11 (p11, exon 50) bases from the 5′ end of the guide strand of the shRNA to ensure selective cleavage of the target sequence [31]. All the exons selected correspond to the 3′ part of the *HTT* mRNA. As the transcript (>10 kb) is too long for the entire sequence to be inserted into lentiviral vectors, we created a chimeric *HTT* gene to test the efficacy and selectivity of our shSNPs. The chimeric HTT contains a N-terminal fragment of mutant HTT corresponding to the first 171 amino acids with 82 glutamines (Htt171-82Q) fused in frame to the part of the protein encoded by the exons carrying the SNP of interest (**Fig. 1A** and Materials and Methods S1). A sequence encoding the HA tag was added to 3′ the end to facilitate detection of the resulting fusion proteins.

**Figure 2:**
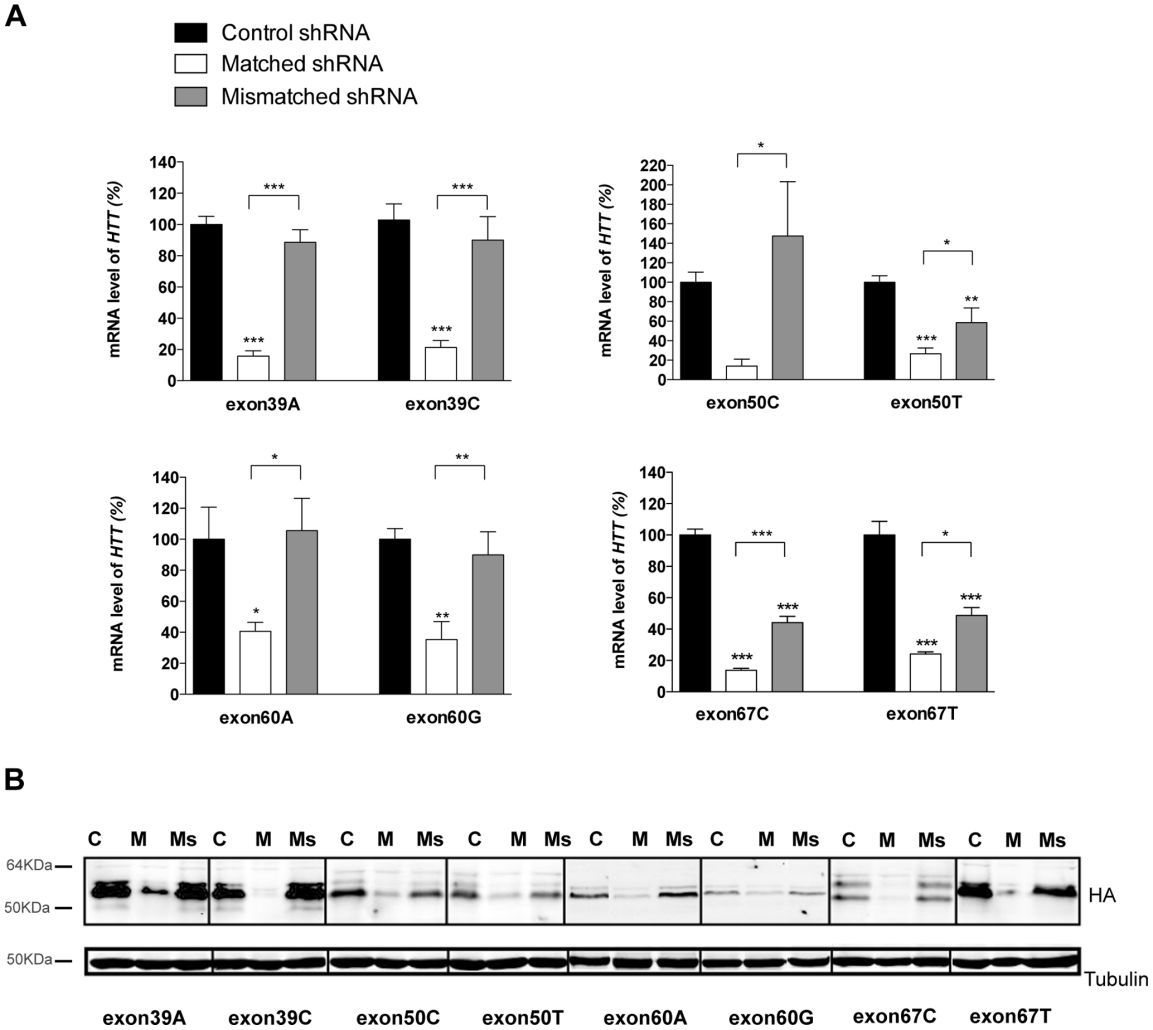
Efficacy and selectivity of the shSNP *in vitro*. (**A**) Quantitative real-time PCR analyses showing the silencing of *HTT* mRNA in transfected 293T cells co-expressing chimeric *HTT* and shSNP or the control shRNA. Levels of the chimeric *HTT* mRNA were normalized to β-Actin and are reported as mean percentages relative to the control condition (set at 100%) ± SEM (n = 3–5). One-way analyses of variance (ANOVA) were performed for each SNP. Newman-Keuls Post-hoc comparison between the shCtrl groups and shSNP groups indicated significant efficacy for sh39A, sh39C, sh50T, sh67T, sh67C (***P<0.001), sh60G (**P<0.01) and sh60A (*P<0.05) whereas the difference between the control condition and the sh50C condition was not statistically significant. The mismatched shRNA conditions were not significantly different from the control condition except for 50T+sh50C (**P<0.01), 67T+sh67C and 67C+sh67T (***P<0.001) and they were all significantly different from the matched shRNA conditions showing the selectivity of this approach *in vitro* (sh39A, sh39C, sh67C (***P<0.001), sh60G (**P<0.01), sh50C, sh50T, sh60A and sh67T (*P<0.05). (**B**) Representative western blot (n = 3) with anti-HA antibody illustrating production of the chimeric proteins. The efficacy test lanes (matched shRNA: M) evidence the decrease/absence of the corresponding chimeric proteins, whereas in selectivity lanes (mismatched shRNA: Ms) the mutant HTT is still present as in the control condition (c).

We tested the ability of the lentiviral vectors encoding the shSNPs to silence their corresponding chimeric constructs. 293T cells were co-transfected with the plasmids expressing the chimeric HTT and the shRNA, and mutant *HTT* levels were analyzed by quantitative RT-PCR (**Fig. 2a**) and western blotting (**Fig. 2B**). The presence of fully matched shSNP was associated with robust degradation of the chimeric *HTT* mRNA (**Fig. 2A**), with the shSNP targeting exons 39 and 67 being the most potent (around 80% of degradation of the targeted mRNA). In contrast, *HTT* mRNA levels were only modestly reduced (10% of silencing in most cases) following co-transfection with mismatched shSNP (**Fig. 2A**). This demonstrates the capacity of this approach to discriminate between the two *HTT* alleles. Selectivity was however poorer for sh50T and sh67C/T, with 40–50% degradation of *HTT* mRNA with the mismatched shRNAs. Additional experiments (n = 3) were performed to demonstrate the correlation between the *HTT* mRNA and protein levels (Figure S1). HTT protein levels were decreased in all matched and, to a lower extent, in most mismatched conditions. A strong correlation was observed between the *HTT* mRNA and protein levels, in agreement with published studies [32], [33].

### Effective allele-specific silencing of mutant *HTT* in the HD rat model

**Figure 3:**
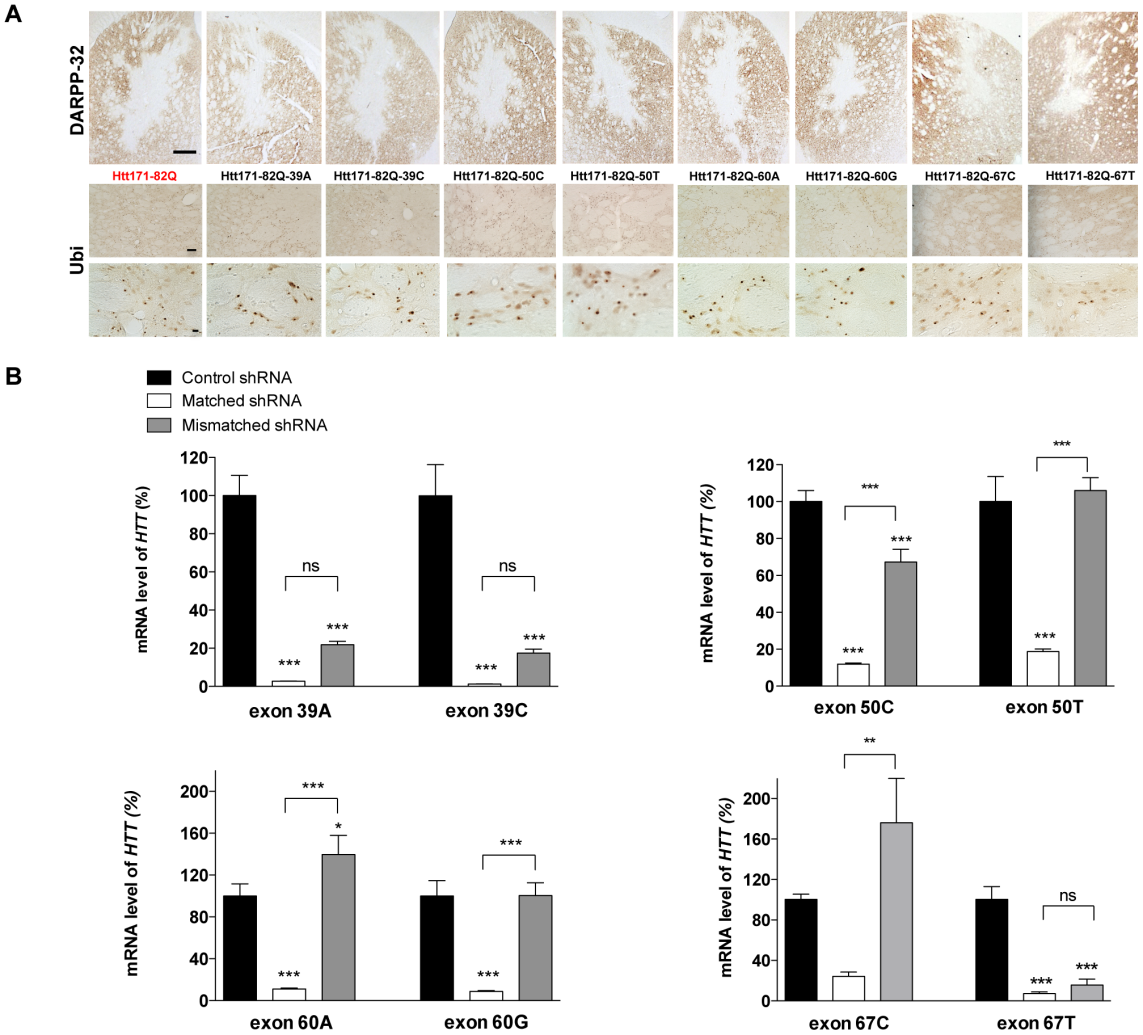
Expression of *HTT* after co-injection of constructs encoding chimeric mutant *HTT* and GFP-shSNPs *in vivo*. (**A**) Lentiviral vectors expressing the htt171-82Q fragment or the various chimeric mutant *HTT* were injected into the striatum of rats (n = 4 per group). Eight weeks after injection, DARPP-32 and ubiquitin (Ubi) staining (low and high magnification pictures) demonstrated that all the constructs led to HD-like neuropathology (loss of DARPP-32 staining and ubiquitin-positive aggregates), similar to that with the htt171-82Q lentiviral-based model. (**B**) Four weeks after injection, the GFP-positive area was laser-capture microdissected and *HTT* mRNA was assayed by RT-qPCR. Values for *HTT* mRNA were normalized to PPIA and are expressed as mean percentages of the value for the control condition ± SEM (n = 6). One-way ANOVAs were conducted for each SNP. 39A: ***P<0.001; 39C: ***P<0.001; 50C: ***P<0.001; 50T: ***P<0.001; 60A: ***P<0.001; 60G: ***P<0.001; 67T: ***P<0.001; 67C: P>0.05. Newman-Keuls Post-hoc comparison between the shCtrl groups and matched shRNA groups demonstrates significant silencing of all targeted *HTT* mRNAs, ***P<0.001, except for exon 67C. Post-hoc comparison between the shCtrl groups and mismatched shRNA groups revealed no significant differences for sh50T, sh60A or sh60G, a significant difference for sh67C, and highly significant differences for sh39A, sh39C, sh50C and sh67T. This comparison as the matched/mismatched post-hoc provides evidence of the different selectivities of the shSNPs.

To evaluate the therapeutic efficacy of this approach *in vivo*, we needed an appropriate model expressing the SNPs of interest. We injected the various chimeric Htt171-82Q-exon vectors and the pathogenic control Htt171-82Q vector into the striatum of adult rats to induce a local expression of the disease protein, and compared the severity of HD neuropathology. Misfolded HTT labeled with the ubiquitin antibody was detected and the typical lesion with a down-regulation of DARPP-32 expression was observed for all the chimeric constructs (**Fig. 3A**), consistent with previous findings [34]. This ubiquitin staining is faithfully reflecting the accumulation of misfolded HTT protein revealed with the EM48 antibody (Figure S2). After 8 weeks, there was no apparent difference between animals injected with the Htt171-82Q or the other Htt171-82Q-exon vectors (**Fig. 3A**).

To assess the efficacy of allele-specific silencing, we co-injected lentiviral vectors encoding human Htt171-82Q-exon with the corresponding shSNP into the striatum of adult rats. Four weeks post-injection, using the GFP-reporter gene present on the vectors expressing the shSNP to identify the infected cells (mostly neurons as previously reported [35] [36]), the GFP-positive area was laser microdissected and the amount of *HTT* mRNA was determined by RT-qPCR. All shSNPs efficiently silenced *HTT* gene expression (**Fig. 3B**). In the same experiment, we then tested the ability of the shRNAs to selectively discriminate SNPs *in vivo*. The striatum of adult rats was co-injected with Htt171-82Q-exon and the corresponding mismatched shRNAs. The shSNPs targeting exons 50 or 60 efficiently discriminated between the two SNP variants (0 to less than 35% degradation of mismatched *HTT* mRNA), whereas the shSNPs targeting exon 39 or 67 did not discriminate (around 80% of degradation) (**Fig. 3B**). These experiments demonstrate the efficiency of all shSNPs is accompanied by good specificity for five of the eight shSNPs.

### Inhibition of HD neuropathology in matched shSNP-treated lentiviral HD rat model

**Figure 4:**
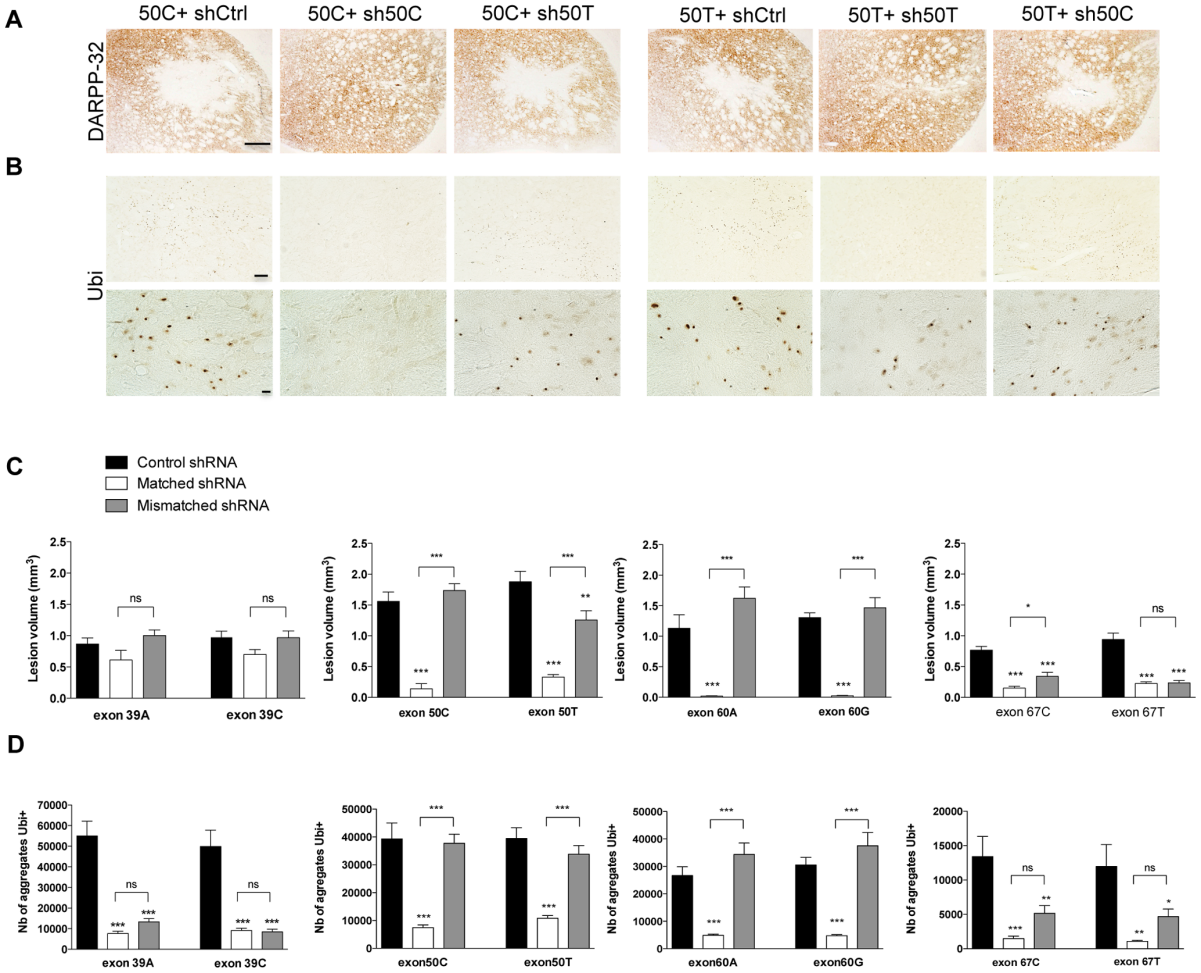
Efficacy and selectivity of the GFP-shSNP *in vivo*. (**A**) and (**C**) DARPP-32 immunostaining and quantification 8 weeks after injection evidencing loss of this striatal marker in the control and mismatched injected areas but its preservation in the matched conditions, except for sh39 and sh67. The results are expressed as mean volumes in mm^3^ depleted of DARPP-32 ± SEM (n = 8). One-way ANOVAs were conducted for each SNP: 50C, 50T, 60A, 60G, 67T, and 67C: ***P<0.001. Newman-Keuls Post-hoc comparison between control conditions and test conditions showed a highly effective prevention of DARPP-32 loss for most of the matched shSNPs and also significant, although less so, prevention of loss for the mismatched sh50C and sh67. For the other mismatched conditions, the post-hoc test was not significant, indicating the selectivity of the shSNPs. (**B**) and (**D**) Ubiquitin immunostaining (low and high magnification pictures) and quantification showing large numbers of aggregates in the control and mismatched injected areas and fewer aggregates in the matched conditions. The results are expressed as mean numbers of ubiquitin-positive aggregates ± SEM (n = 8). One-way ANOVAs were conducted for each SNP: 39A, 39C, 50C, 50T, 60A, 60G, and 67T: ***P<0.001; 67C: **P<0.01. Newman-Keuls Post-hoc comparison between control and matched conditions showed highly significant prevention of aggregate formation for all matched shSNPs. For mismatched conditions, the post-hoc test was not significant except for the mismatched sh39 and sh67.

To further confirm the therapeutic potential of the shSNPs, we investigated the formation of HTT aggregates and striatal neuronal dysfunction. HTT inclusions are a hallmark of the pathology and a reliable marker of the severity of HD pathology. We co-injected the chimeric constructs with either the fully matched or mismatched shSNPs or a shRNA control (shCtrl). Eight weeks later, we quantified the down-regulation of DARPP-32 expression and the number of ubiquitin-positive aggregates. DARPP-32 immunostaining (**Fig. 4A**) showed that the lesion size was reduced in the matched conditions, except for the sh39, but not in control and some mismatch injected areas (**Fig. 4C**). This staining indicated that most of the shSNPs in the matched condition inhibited the appearance of an HD neuropathology with very high efficacy. Large numbers of ubiquitin immunostained aggregates were observed in the control and most mismatch injected areas, whereas smaller numbers were found in the matched conditions (**Figs. 4B and 4D**). In the mismatch condition, the SNP discrimination power of sh39 and sh67 was low (not significant; **Figs. 4C and 4D**). Injection of the shSNP only didn't lead to striatal dysfunction based on DARPP32 staining, 8 weeks after injection (data not shown).

Pfister and collaborators have shown that for the SNP in exon 39, placing the mismatch at position 16 rather than 10 improved the discrimination ratio in a luciferase reporter system [13]. We therefore developed new vectors encoding sh39p16 and compared them with the sh39p10 constructs by evaluating *in vitro* mutant *HTT* mRNA levels in 293T cells co-transfected with the chimeric *HTT* and the shRNA (Figure S3A) and *in vivo* lesion volumes and the numbers of ubiquitin-positive aggregates in co-injected rats (Figure S3B and S3C). Sh39p16 efficiently silenced mutant *HTT*, however the selectivity was not improved by placing the mismatched base at position 16 of the shRNA.

### Efficient silencing of mutant *HTT* in BACHD mice using matched shSNP

**Figure 5:**
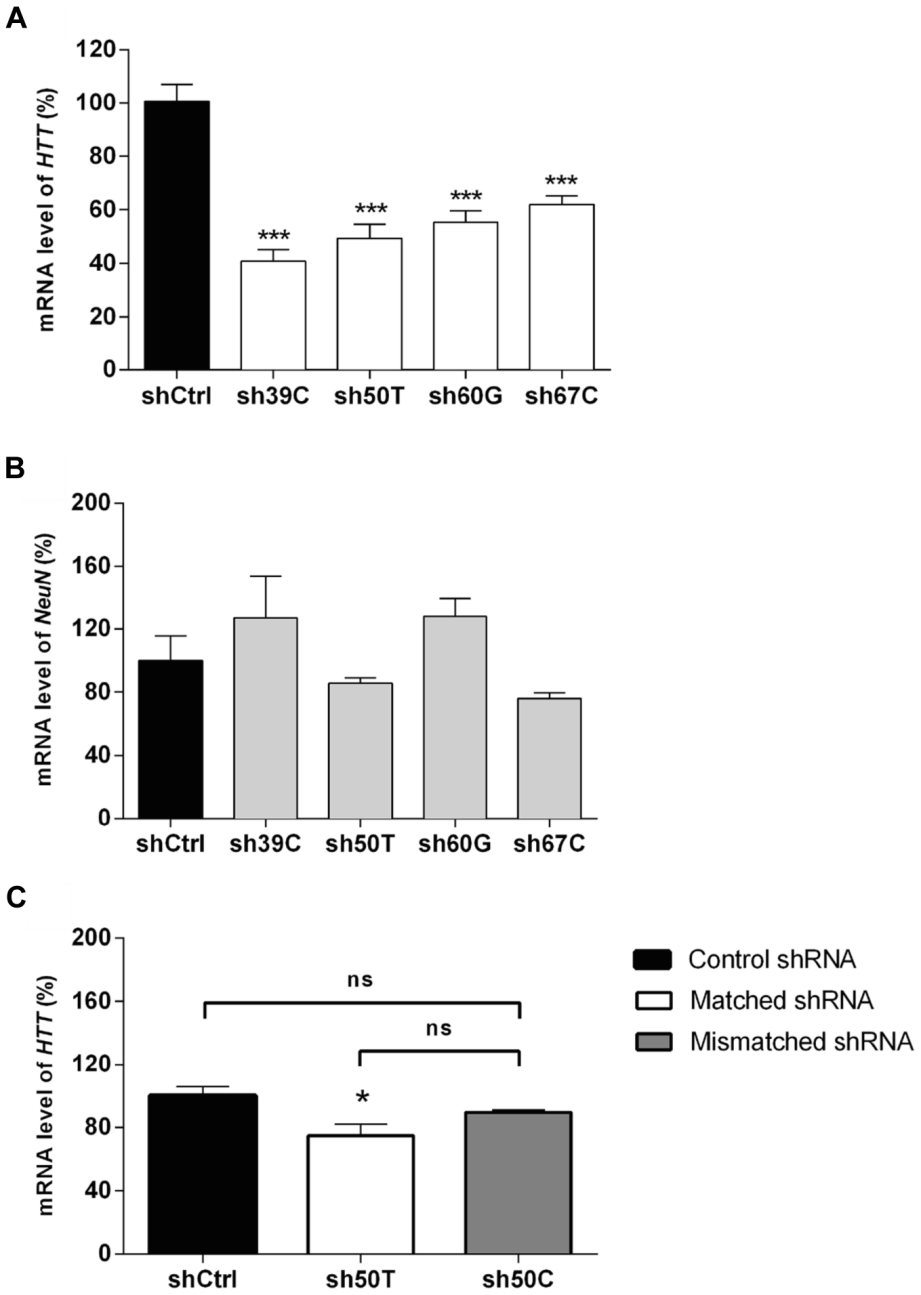
Effective and selective SNP-specific silencing in BACHD mice. (**A**) Four weeks after injection of constructs encoding fully matched shSNP in BACHD mice striatum, the GFP-positive area was dissected and *HTT* mRNA was assayed by RT-qPCR. Values for *HTT* mRNA were normalized to a reference peptidyl propyl isomerase A (*PPIA*) and are expressed as mean percentages relative to the control condition (set at 100%) ± SEM (n = 10). One-way ANOVAs were conducted for each matched SNP for *HTT* 39C, 50T, 60G, and 67C: ***P<0.001. Newman-Keuls Post-hoc comparison between the shCtrl and matched shRNA groups demonstrate significant silencing of targeted human *HTT* mRNA. (**B**) Samples all showed similar NeuN gene expression (One-way ANOVAs and Newman-Keuls Post-hoc comparison test). (**c**) Three weeks after injection of constructs encoding shCtrl, sh50T and sh50C in BACHD mice striatum, 1 mm^3^ punches of the GFP infected area were dissected and *HTT* mRNA was assayed by RT-qPCR with primers specific for the human *HTT*. Values for *HTT* mRNA were normalized to a reference *PPIA* and are expressed as mean percentages relative to the control condition (set at 100%) ± SEM (n = 4–6). One-way ANOVAs were conducted for each groups: *P<0.05. Newman-Keuls Post-hoc comparison between the shCtrl and the sh50T confirm the silencing of human mutant *HTT* mRNA, whereas no statistically significant difference was observed between shCtrl and sh50C groups, demonstrating the selectivity of *HTT* silencing.

BACHD mice express full-length human mutant *HTT* at physiological levels. Human mutant *HTT* mRNA in these mice is approximately 1.36 fold the level of endogenous WT *HTT* [37]; whereas mutant *HTT* mRNA level in animals injected with lentiviral vectors is about 25 times higher than the endogenous one [5]. We therefore used these animals to measure silencing efficacy on endogenous full-length *HTT* mRNA in the disease context. Genotyping showed that these mice express exon39C, exon60G and exon67C, and exon50T in agreement with Carroll et al. [15]. The four matched shSNPs and the shCtrl were injected bilaterally into the striatum of these adult and pre-symptomatic transgenic mice (we chose to inject sh39Cp16 because it is more efficacious than sh39Cp10, Figure S3). Four weeks post-injection, the mice were sacrificed and the GFP-positive area of the striatum was grossly punched out from 1 mm-thick striatal slices. *HTT* mRNA was measured by RT-qPCR from these punches to measure the average silencing obtained in the striatum (i.e. infected/treated and non-infected cells) and evaluate treatment efficiency, which can be expected with this gene transfer approach. The data gathered from this *in vivo* experiment and those presented in Figures 3–4 are therefore complementary and provide two distinct types of information on the allele-specific silencing. In striatal punches from BACHD mice, the amounts of mutant *HTT* mRNA (**Fig. 5A**) were lower (around 50%) in mice treated with each of the four shSNPs than shCtrl. There was no significant difference in the mRNA levels of the neuronal marker Neuronal Nuclei (NeuN) between any of the samples (**Fig. 5B**) confirming that they were comparable. To further demonstrate the selectivity of the silencing on full-length human mutant *HTT*, we performed one additional experiment in BACHD mice injected with sh50T, sh50C and shCtrl. As expected, a statistically significant silencing was observed with the sh50T while *HTT* mRNA level in the sh50C group was similar to the control group (**Fig. 5C**). Therefore, the capacity to discriminate the two alleles was confirmed.

### Functional recovery after allele-specific silencing of mutant *HTT* in neurons derived from HD-hESCs

**Figure 6:**
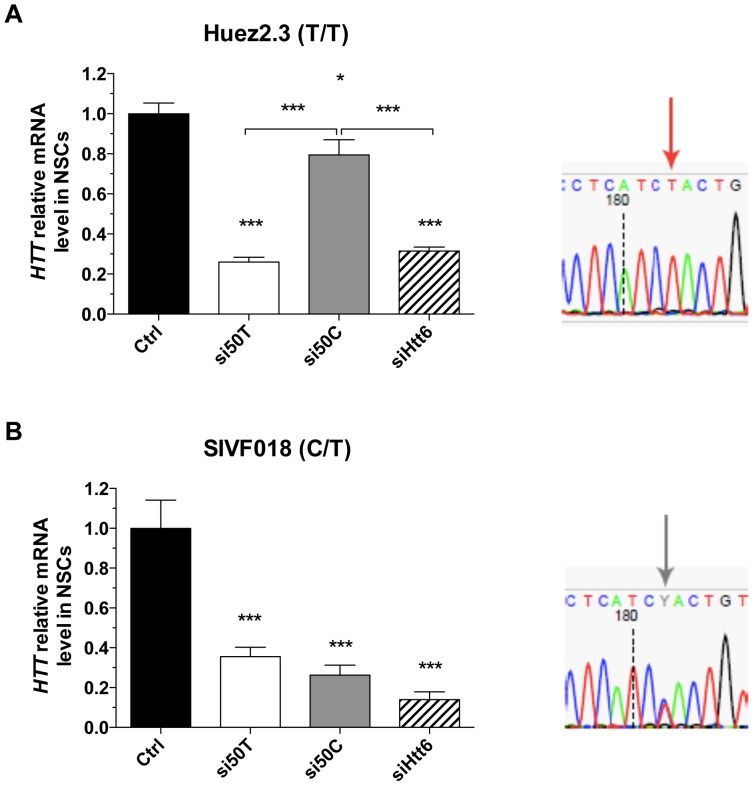
Allele specific knock-down of *HTT* mRNA in human HD NSC. Relative *HTT* mRNA levels normalized to controls (non-targeting siRNA) measured by RT-qPCR in HD-NSCs transfected with pan-allelic (siHtt6) or allele-specific *HTT*-targeting (si50T and si50C) siRNAs. HD-NSCs were derived from two HD-hESC lines, Huez2.3 (**A**) and SIVF018 (**B**), T/T homozygous and C/T heterozygous for SNP rs362331, respectively. n =  4. P-value by one-way ANOVA and Tukey's multiple comparison test; *P<0.05; **P<0.01; ***P<0.001; Error bars depict SEM.

To assess the silencing of endogenous *HTT* and the functional recovery, we took advantage of the recent isolation of human neural stem cells (NSCs) from HD-hESCs [26]. We transfected the HD-NSC line Huez2.3 homozygous for the SNP in exon 50 (T/T) and demonstrated that the silencing obtained with si50T was similar to that with the previously described siHtt6, which targets both alleles [5]. Transfecting the cells with the mismatched siRNA (si50C) had little effect on *HTT* expression, confirming the selectivity of the approach (**Fig. 6A**). Because the CAG expansion is located far from the SNP position, we then developed a new method based on SNP-specific reverse transcription and amplification of CAG repeats by PCR, coupled with size detection on a chip, to link the SNP and the mutant allele in the heterozygous HD-hESC. Using this strategy, we found that the mutant CAG expansion (46 or 44 CAG) was associated with the SNP50T in SIVF018 and VUB05 cells. Treatment of these cells with si50T and si50C significantly decreased mutant and WT *HTT* mRNA levels, respectively, and as expected the silencing was more pronounced with siHtt6 (**Fig. 6B**).

**Figure 7:**
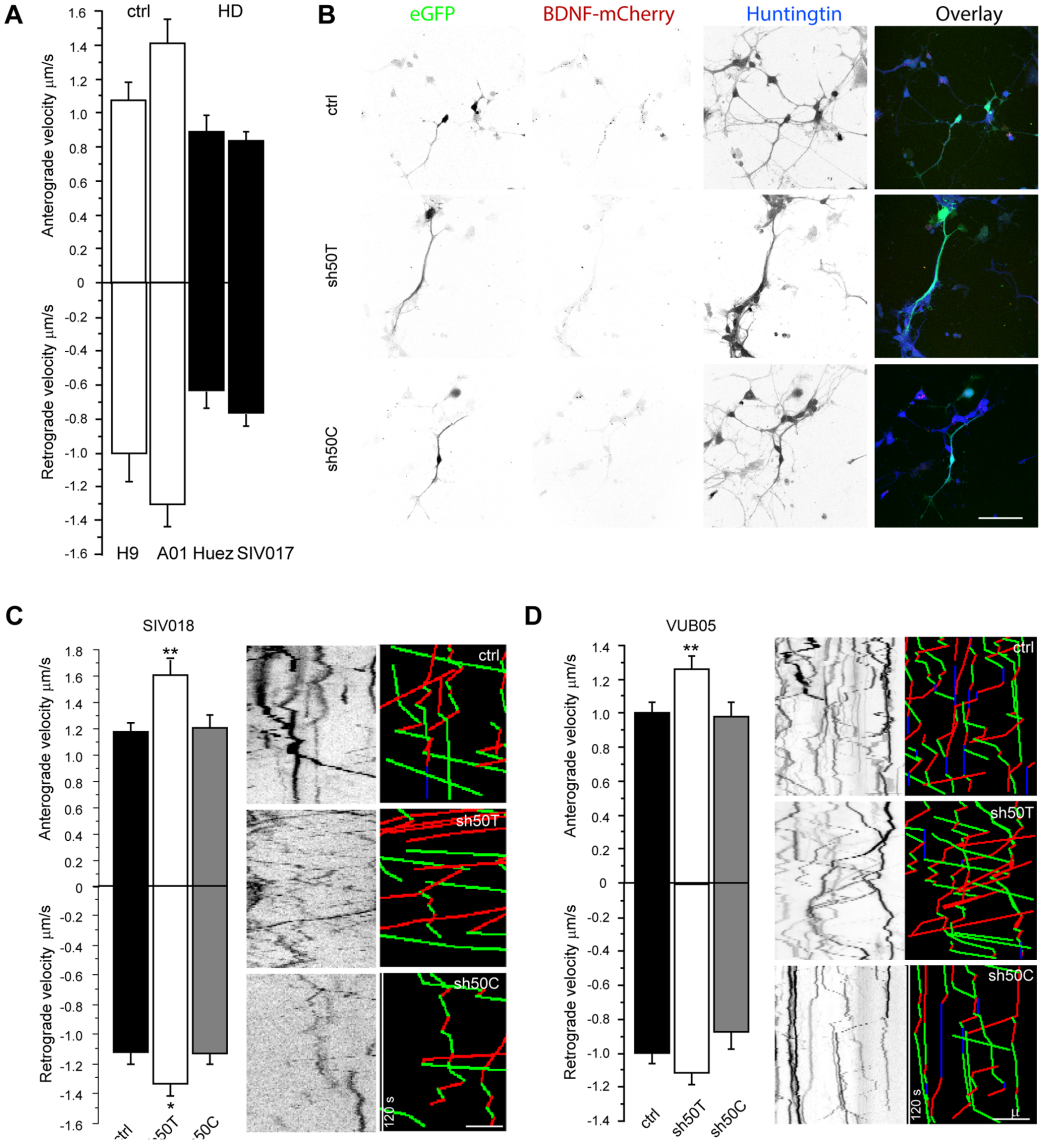
Recovery of BDNF trafficking after allele-specific silencing of mutant *HTT* in neurons derived from HD-hESCs. (**A**) Quantitative analyses of anterograde and retrograde BDNF vesicular velocity in two different WT NSC lines (H9 and SA-01; white bars) and two HD-derived NSC lines (Huez2.3, SIVF017; black bars): anterograde and retrograde velocities are higher in WT neurons than in HD cells. One-way ANOVAs were conducted for anterograde and retrograde velocity separately. Anterograde: ***P<0.001; n = 43 to 170. Retrograde: **P<0.01; n = 43 to 133. (**B**) Direct GFP and mCherry fluorescence and immunofluorescence staining of HTT in transfected neurons derived from one HD-hESC line (VUB05); these cells were used for video-microscopy analyses. (**C, D**) Quantitative analyses of anterograde and retrograde BDNF vesicular velocities in SIVF017 cells (**C**) and VUB05 cells (**D**) with representative kymographs and the analyzed trajectories (green for anterograde, red for retrograde and blue for pausing vesicles). (**C**) One-way ANOVA for anterograde velocity: **P<0.01; n = 50 to 72. Fisher's PLSD Post-hoc test demonstrated significant velocity recovery with respect to the control group in the sh50T group, **P<0.01, but not in the sh50C group. For retrograde: F(2,198) = 3.095, *P<0.05; 55 to 77 events were recorded for each group. Fisher's PLSD Post-hoc test demonstrated significant velocity recovery with respect to the control group in the sh50T group, *P<0.05 but not in the si50C group. (**D**) One-way ANOVAs were conducted for anterograde and retrograde velocity separately. For anterograde: F(2, 329) = 5.494, **P<0.01; 51 to 153 events were recorded for each group. Fisher's PLSD Post-hoc test demonstrated a significant increase in velocity for the sh50T group, **P<0.01 but not the sh50C group, with respect to the control group. For retrograde: F(2,313) = 2.585, P = 0.077; 44 to 144 events were recorded for each group. There was no significant difference between control group and treated groups for retrograde transport.

We tested whether the allele-specific silencing of mutant *HTT* was associated with a functional recovery. We took advantage of the defect in the vesicular trafficking of the brain-derived neurotrophic factor, BDNF, previously described in primary cultures expressing mutant HTT [30]. We measured BDNF anterograde and retrograde velocity in neurons derived from WT (lines H9 and SA-01) and HD lines (Huez2.3 and SIVF018) (**Fig. 7A**). Direct fluorescence allowed us to select cells co-transfected with BDNF-mCherry and shRNA (GFP) (**Fig. 7B**). Kymographs showing the spatial positions of BDNF vesicles through time were used to quantify the dynamics in different conditions (**Figs. 7C and 7D**). When differentiated into neurons, both HD-lines (Huez2.3 and SIVF018) displayed vesicular trafficking defects relative to the WT lines (H9 and SA-01) (**Fig. 7A**). This confirms that an expansion as limited as 44 CAG is sufficient to impair transport function [30]. Immunofluorescence staining confirmed that these cells expressed HTT (**Fig. 7B**). We then analyzed mCherry-BDNF transport in the SIVF018 line and a second HD heterozygous line, VUB05. Both anterograde and retrograde trafficking of BDNF was significantly greater in SIVF018 and VUB05 neurons when the mutant *HTT* was silenced with the sh50T, whereas targeting the WT *HTT* allele with sh50C had no effect (**Figs. 7C and 7D**). Thus, allele-specific silencing of mutant *HTT* in HD cells is associated with a functional recovery of BDNF transport. Also, silencing of the mutant allele is sufficient to alleviate the dominant effect of the mutant *HTT* over the WT *HTT*.

## Discussion

Targeting heterozygous SNPs to degrade mutant *HTT* selectively while preserving the WT transcript is an extremely promising approach to HD treatment. However, developing and validating appropriate protocols is challenging. The frequencies and maps of heterozygous SNPs in the population have been established [13]–[18], and the number of SNP candidates is limited (approximately 40–50) compared to the countless sequences that could be targeted with a non allele-specific strategy. Also, the sequences flanking an SNP are not necessarily optimal for shRNA interaction, and the mismatches in SNPs have different discrimination powers that affect the selectivity of the silencing further reducing the number of possibilities. Furthermore, clinical implementation will undoubtedly require the development and validation of several products to treat the most patients.

Here, we addressed some of these issues by targeting four SNP among the most frequent in the population. We showed that the eight shRNAs covering these SNPs provide efficient and, in most cases, selective silencing of *HTT in vitro* and *in vivo* and hence are good candidates for the treatment of many HD patients.

These findings were obtained with novel models developed specifically for screening shSNPs. The chimeric reporter plasmids expressing the construct encoding human mutant *HTT* fused to the exons containing the various SNP allow large-scale and quick quantitative analysis of the silencing efficacy and selectivity. We reported that the allele-specific silencing demonstrated in 293T cells was reproduced in human NSC derived from HD-hESC lines involving a mutated *HTT* corresponding to that responsible for adult onset HD. These experiments showed that the *in vitro* platform based on 293T cells and HD-specific human pluripotent stem cells is suitable for evaluating the effects of allele-specific shRNA. We reported that neurons derived from HD-hESCs reproduce the defect in anterograde and retrograde BDNF vesicular transport associated with HD [30]. Allele-specific silencing of mutant *HTT* restored the mean velocity of BDNF vesicles to control values. Silencing of WT *HTT* in neurons heterozygous for SNP exon 50 did not alter trafficking along microtubules in neurites, as previously reported [38]. The different effects of treatment with sh50T and sh50C suggest that these shSNP are sufficiently potent and selective to modulate axonal vesicular transport in neurons. Importantly, *HTT* mRNA levels in NSC derived from hESC are closer of the values observed in patient brain samples than in HD-derived fibroblasts or lymphoblasts (which are 100x lower), increasing the potential value of this cellular model [26].

Silencing levels were similar to those obtained with shRNA targeting both alleles [5]. Even though the sequences of these shSNP were not selected according to established algorithms and criteria for optimal silencing, all of them efficiently promoted degradation of the target mRNA. In agreement with Lombardi et al. [14], we observed that the sh50C/T led to efficient degradation of the targeted mRNA (74%; fully matched sequence), while a central mismatch in the shRNA sequence abolished this silencing (21%; mismatched sequence). In contrast, antisense oligonucleotides (ASO) targeting the exon 50 were associated with poor discrimination power in human HD fibroblasts [15]. This discrepancy might be due to differences in the mode of action between shRNA and ASO, the experimental paradigm, method of administration modalities or different concentrations.

HD transgenic mice available when we initiated this study, express only one allele of the human *HTT* gene and only full-length *HTT* genes are suitable for validating the chosen SNP, most of which map in the 3′ part of the gene. We therefore used two approaches for *in vivo* validation. As a first screen to assess the efficacy and selectivity of *HTT* allele-specific silencing, we injected lentiviral vectors encoding the first 171 amino acids of the human mutant *HTT* fused to the exon containing the SNP into the striatum of adult rats [5], [34]. Our shSNP recognized only the human *HTT* transcript, because the targeted regions display only limited sequence identity with the rodent *HTT* mRNA. These experiments therefore replicate allele-specific silencing as it would occur in human patients. Four weeks after the injection, the majority of the animals treated with fully matched shRNA showed *HTT* mRNA levels that were 80% lower than control values, and a concomitant reduction of the number of *HTT* aggregates and lesion size at 8 weeks. These experiments confirmed the efficacy and selectivity of the shSNPs targeting Exon50C/T and Exon60G/A. The shSNPs specific for exon 39A/C and exon 67C/T were also extremely effective, although their discriminatory power was not completely preserved. Additionally, besides a strong transgene knockdown by targeting exon 39, as shown by reduction of target RNA and ubiquitin inclusions, there was a limited rescue of the lesion volume. These particular shRNAs could be partially complementary to one or several rat's transcripts and lead to their expression alteration, ultimately causing toxicity. Indeed, Jackson and collaborators [39] reported that 11 consecutive nucleotides could lead to unspecific degradation of off-target mRNA. However, in a human context, the panel of potential targets may be different because of sequence dissimilarities between rodents and human, therefore the shSNP39 could be better tolerated. Unfortunately, we are unable to verify this hypothesis since none of our human neural stem cell lines are heterozygous for SNP39; treating with either shSNP39 would degrade the WT *HTT* allele, which would provoke transcriptomic changes [6]. Work in Aronin's lab suggests that placing the SNP exon 39 at p16 of the shRNA might be more favorable than at p10 to discriminate the two alleles [13]. We tested this *in vivo* and found no improvement in selectivity but, interestingly, the DARPP-32 lesion volume was significantly smaller indicating a better tolerance of this sequence. These experiments demonstrate that most of the shSNP can selectively reduce the expression of the disease-causing *HTT* allele without significantly inhibiting expression of the WT allele *in vitro* or *in vivo*. To assess silencing of the endogenous full-length *HTT* transcript, we injected the lentiviral vectors into the striatum of BACHD mice. BACHD mice have alleles which are present in most HD patients, whereas this is not the case for YAC128 [15]. SNP in exons 39 and 50 are in linkage disequilibrium with each other and with the CAG expansion, and there are no such associations for the SNPs in exon 60 and 67 [14]. RT-qPCR analysis confirmed that all shRNAs targeting the SNPs present in these animals led to efficient degradation of the full-length mutant human *HTT*. It is important to mention that the SNPs 67C/T were in their original context here (i.e. 3′UTR) whereas they were in the coding sequence in the lentiviral HD rat model. Anyhow, it didn't alter its ability to trigger RNA cleavage as showed by decreased levels of *HTT* mRNA in both models. Nevertheless, we can't exclude that part of this effect is mediated by translational repression or mRNA decay mediated by Ago1, 3 and 4 as previously demonstrated [40], [41].

The clinical implementation of this type of strategy will require the genotyping of HD patients for the selected SNPs, and, to treat a large proportion of HD patients, the development of eight therapeutic products (shSNP). A growing number of pre-clinical and clinical trials have been launched to assess the use of RNAi for the treatment of autosomal dominant diseases [42]. Both exogenous oligonucleotides and shRNA produced by the target cells are being investigated. Chemically modified siRNAs and ASO have an excellent safety profile and half-life, but the issues of crossing the blood-brain barrier to reach target cells in deep brain areas and long-term administration in general still represent major challenges for these therapeutic agents. Intraventricular infusion allows broad diffusion throughout the brain, but the dose of oligonucleotides needed to achieve efficient silencing are very high and could lead to an oversaturation of the RNAi machinery at the level of the RISC complex [9], [43]. An alternative route of entry into the CNS is intraparenchymal administration of appropriate viral vectors. The design, production, and efficiency of gene transfer vectors, especially for transduction of the central nervous system (CNS), has improved remarkably, and protocols for safe transduction and long-term and robust transgene expression in the brain are now available [21], [44]-[46]. These developments have led to the initiation of several phase I/II clinical trials with adeno-associated vectors (AAV) [47]; more recently trials have started with lentiviral vectors for the treatment of adrenoleukodystrophy [48] and Parkinson's disease [49]. The issue of the safety of shRNA (off target effects) and lentiviral vectors (genotoxicity profile) needs further evaluation. Nevertheless, our study and the rapidly accumulating data evidencing the potential value of this type of approach argue for continued efforts to develop clinical applications of these techniques.

## Supporting Information

Figure S1 **Correlation between** ***HTT*** **mRNA and protein levels in 293T transfected cells.** (**A**) Levels of the chimeric HTT, represented by HA (representative pictures Figure 2B), were normalized to tubulin and are reported as mean percentages relative to the control condition (set at 100%) ± SEM (n = 3). One-way analyses of variance (ANOVA) were performed for each SNP. Newman-Keuls Post-hoc comparison was conducted between the control shRNA and matched shRNA (***p<0.001: 39A, 50C, 60A, 60G, 67C, 67T; **p<0.01: 50T) and between the matched shRNA and mismatched shRNA (***p<0.001: 39A, 60A, 67C, 67T; **p<0.05: 60G). (**B, C**) Distribution of *HTT* RT-qPCR and western blot data (n = 3) normalized on shUNIV results in 293T cells obtained with all match (**B**) or mismatch (**C**) shRNAs. Results demonstrate a high correlation between the *HTT* mRNA and proteins quantities independently of the exon targeted (for match shRNA: r = 0.63, p<0.01; for mismatch shRNAs: r = 0.66, p<0.001).
(TIF)

Figure S2 **Number of Ubiquitin-positive aggregates is highly correlated to the number of HTT aggregates.** (**A**) and (**B**) EM-48 immunostaining and quantification showing large numbers of aggregates in the control and mismatched injected areas and fewer aggregates in the matched conditions for the SNP 50C and 50T (same animals as in **Fig. 4**). The results are expressed as mean numbers of EM-48-positive aggregates ± SEM (n = 6–7). One-way ANOVAs were conducted for each SNP; 50C and 50T: ***P<0.001. Newman-Keuls Post-hoc comparison between control and matched conditions showed highly significant prevention of aggregates formation for the matched shSNPs. For mismatched conditions, the post-hoc test was not significant compared to control condition. Matched and mismatched conditions were significantly different for the 2 SNP studied (50C: ***p<0.001; 50T: **p<0.01). (**C**) Correlation between the numbers of Ubiquitin- and EM-48-positive aggregates quantified from the same animals. EM-48-positive aggregates are always more numerous but the number is highly correlated to the number of ubiquitin-positive aggregates (Bravais-Pearson correlation coefficient r = 0.93; p<0.001).
(TIF)

Figure S3 **Efficacy and selectivity of the sh39A and sh39C with SNP in p10 or p16.** (**A**) Quantitative real-time PCR analyses showing the silencing of *HTT* mRNA in transfected 293T cells co-expressing chimeric *HTT* and shSNP or the control shRNA. Levels of the chimeric *HTT* mRNA were normalized to a reference peptidyl propyl isomerase A (*PPIA*) and are expressed as mean percentages relative to the control condition (set at 100%) ± SEM (n = 3). One-way ANOVAs were performed for each SNP. Newman-Keuls Post-hoc comparison between the shCtrl and shSNP groups indicated significant efficacy for the two SNP in the two positions: sh39Ap10, sh39Ap16 (*P<0.05), sh39Cp10 and sh39Cp16 (**P<0.01). The mismatched sh39p16 conditions were significantly different from the control condition (sh39Ap16 (*P<0.05) and sh39Cp16 (**P<0.01)). (**B**) and (**C**) Eight weeks post-injection, DARPP32 and ubiquitin immunoreactivity were used to quantify the lesion volume and the number of ubiquitin-positive aggregates after sh39p10 or sh39p16 co-injection with htt171-82-exon39. The results are expressed as means ± SEM (n = 8). One-way ANOVAs were conducted for each SNP (**P<0.01; ***P<0.001). Newman-Keuls post-hoc comparison between control and matched shRNA groups concerning the size of the lesion showed significant efficacy for the shSNP only in position 16 (**B**). Regarding the number of aggregates (**C**), the four constructs were effective. However, for the mismatched conditions, the post-hoc test also revealed significant differences for both stainings revealing that these shSNP are not selective.
(TIF)

Table S1 **Sequences of the primers used to generate fragments of** ***HTT*** **gene containing each SNP.** The sequences targeted by the shSNPs are in bold. The position of the SNP is in red.
(DOC)

Table S2 **Sequences of the oligonucleotides used to generate the shRNA targeting the SNP and the controls.** The sense and anti-sense strands of the shRNA are given in bold. The position of the SNP is in red.
(DOC)

Table S3 **Sequences of the primers used for RT-qPCR.**
(DOC)

Table S4 **List of primer sequences used for SNP sequencing in NSC.**
(DOC)

Materials and Methods S1 (DOCX)
