# Supplementary figures and images for: Allele-Specific Silencing of Mutant Huntingtin in Rodent Brain and Human Stem Cells
Source: PLoS One. 2014 Jun 13;9(6):e99341. doi: 10.1371/journal.pone.0099341 (PMC4057216; doi:10.1371/journal.pone.0099341)

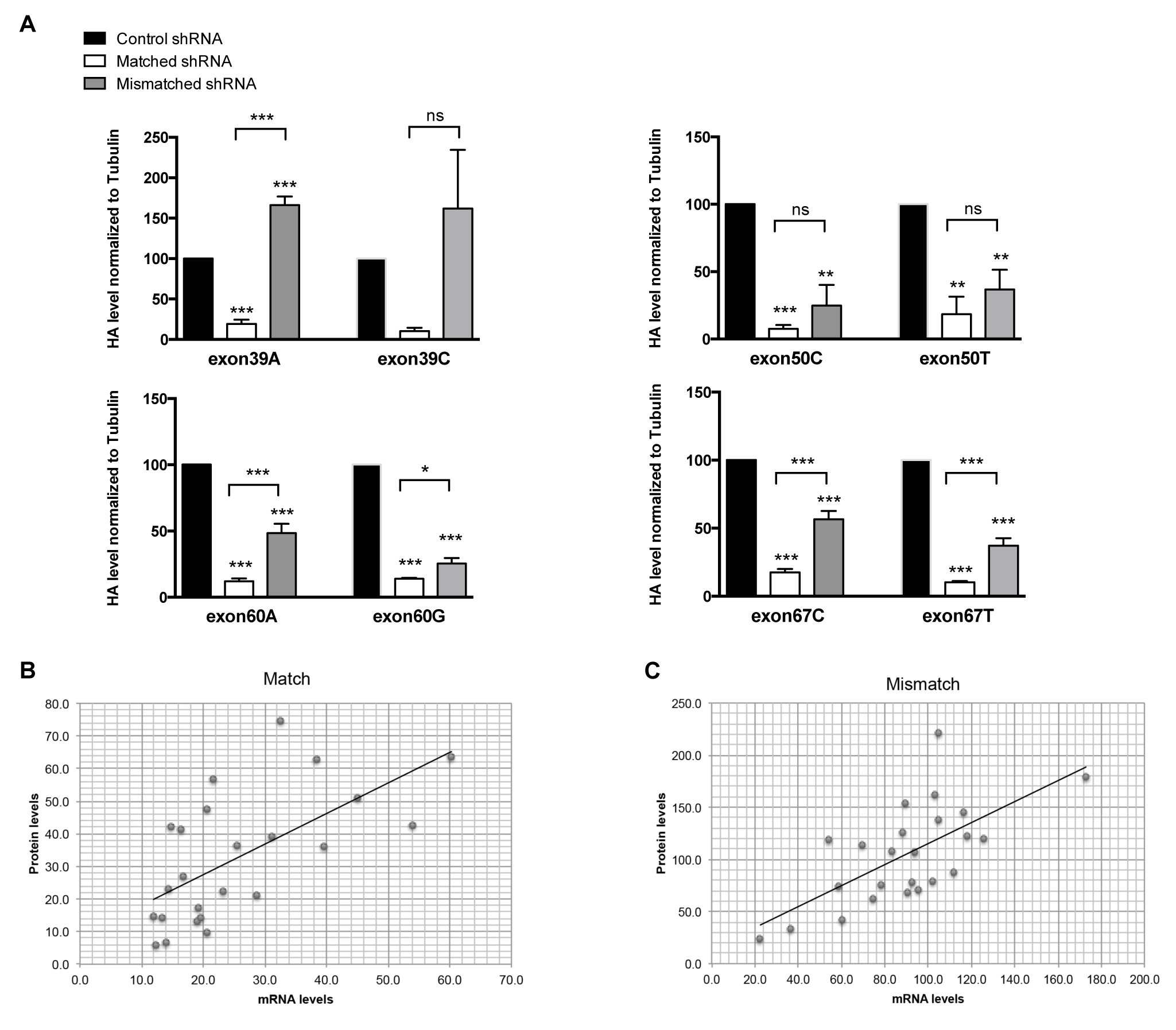

Supplement: Figure S1 — Correlation between HTT mRNA and protein levels in 293T transfected cells. (A) Levels of the chimeric HTT, represented by HA (representative pictures Figure 2B), were normalized to tubulin and are reported as mean percentages relative to the control condition (set at 100%) ± SEM (n = 3). One-way analyses of variance (ANOVA) were performed for each SNP. Newman-Keuls Post-hoc comparison was conducted between the control shRNA and matched shRNA (***p<0.001: 39A, 50C, 60A, 60G, 67C, 67T; **p<0.01: 50T) and between the matched shRNA and mismatched shRNA (***p<0.001: 39A, 60A, 67C, 67T; **p<0.05: 60G). (B, C) Distribution of HTT RT-qPCR and western blot data (n = 3) normalized on shUNIV results in 293T cells obtained with all match (B) or mismatch (C) shRNAs. Results demonstrate a high correlation between the HTT mRNA and proteins quantities independently of the exon targeted (for match shRNA: r = 0.63, p<0.01; for mismatch shRNAs: r = 0.66, p<0.001). (TIF) [file pone.0099341.s001.tif]

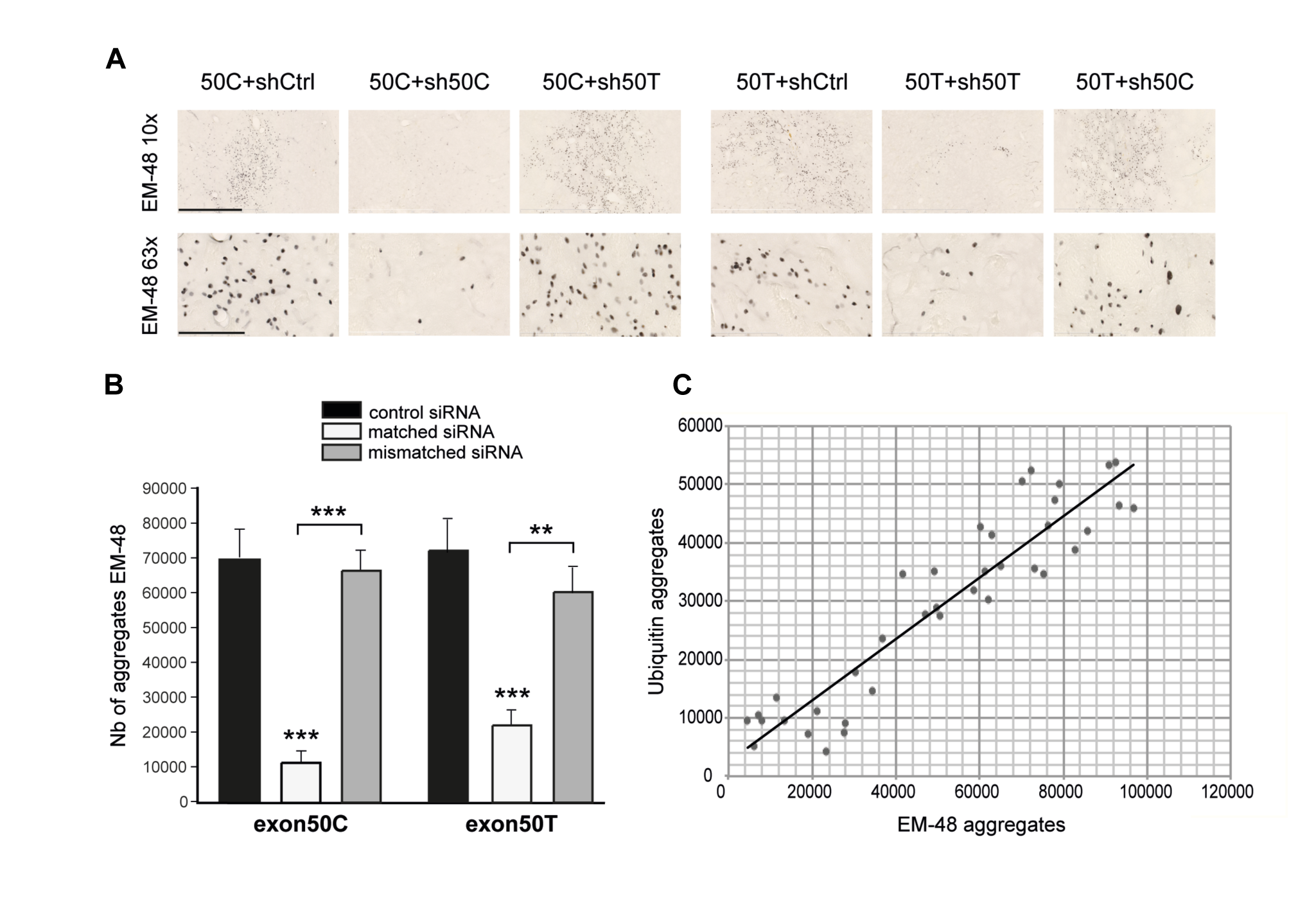

Supplement: Figure S2 — Number of Ubiquitin-positive aggregates is highly correlated to the number of HTT aggregates. (A) and (B) EM-48 immunostaining and quantification showing large numbers of aggregates in the control and mismatched injected areas and fewer aggregates in the matched conditions for the SNP 50C and 50T (same animals as in Fig. 4 ). The results are expressed as mean numbers of EM-48-positive aggregates ± SEM (n = 6–7). One-way ANOVAs were conducted for each SNP; 50C and 50T: ***P<0.001. Newman-Keuls Post-hoc comparison between control and matched conditions showed highly significant prevention of aggregates formation for the matched shSNPs. For mismatched conditions, the post-hoc test was not significant compared to control condition. Matched and mismatched conditions were significantly different for the 2 SNP studied (50C: ***p<0.001; 50T: **p<0.01). (C) Correlation between the numbers of Ubiquitin- and EM-48-positive aggregates quantified from the same animals. EM-48-positive aggregates are always more numerous but the number is highly correlated to the number of ubiquitin-positive aggregates (Bravais-Pearson correlation coefficient r = 0.93; p<0.001). (TIF) [file pone.0099341.s002.tif]

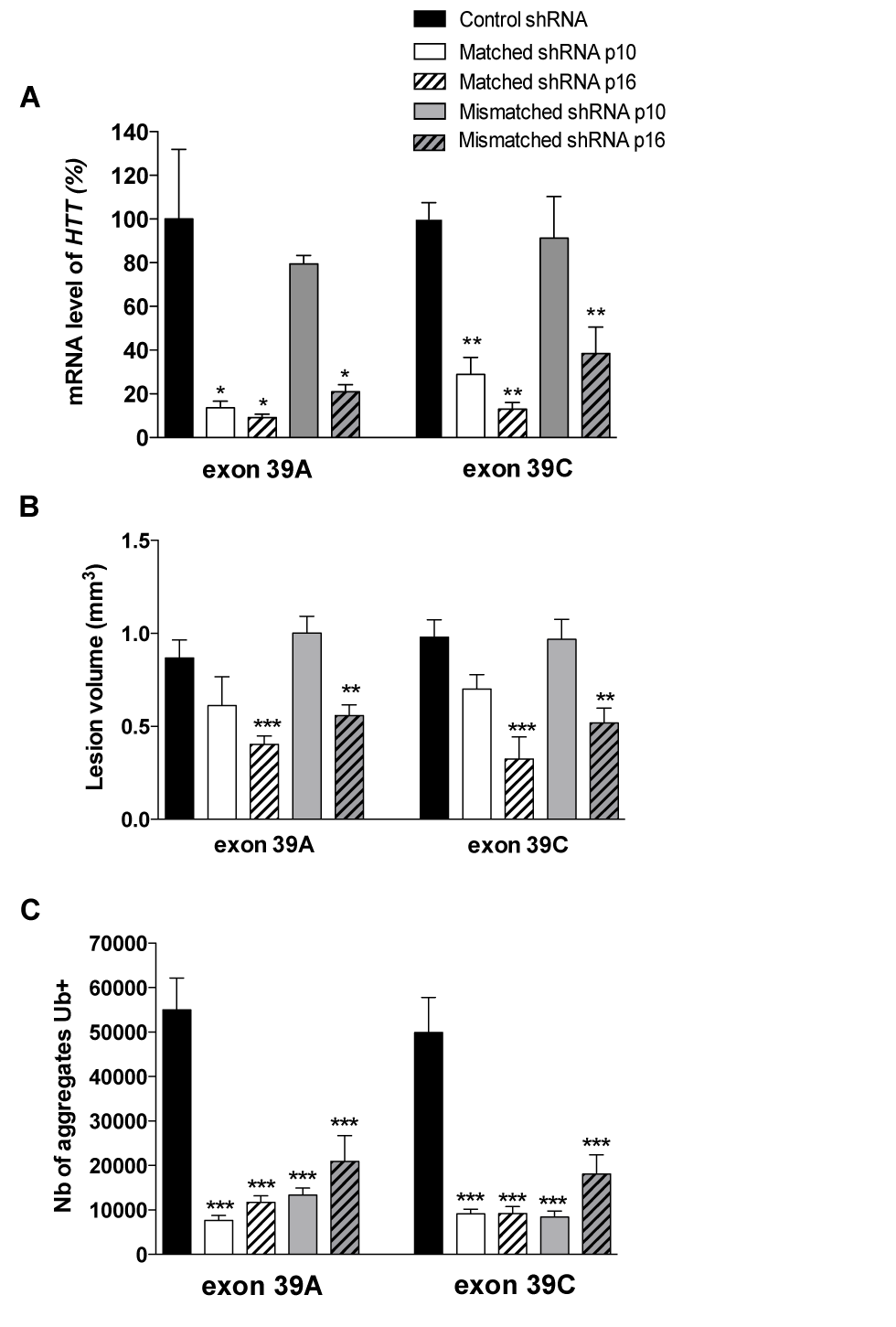

Supplement: Figure S3 — Efficacy and selectivity of the sh39A and sh39C with SNP in p10 or p16. (A) Quantitative real-time PCR analyses showing the silencing of HTT mRNA in transfected 293T cells co-expressing chimeric HTT and shSNP or the control shRNA. Levels of the chimeric HTT mRNA were normalized to a reference peptidyl propyl isomerase A (PPIA) and are expressed as mean percentages relative to the control condition (set at 100%) ± SEM (n = 3). One-way ANOVAs were performed for each SNP. Newman-Keuls Post-hoc comparison between the shCtrl and shSNP groups indicated significant efficacy for the two SNP in the two positions: sh39Ap10, sh39Ap16 (*P<0.05), sh39Cp10 and sh39Cp16 (**P<0.01). The mismatched sh39p16 conditions were significantly different from the control condition (sh39Ap16 (*P<0.05) and sh39Cp16 (**P<0.01)). (B) and (C) Eight weeks post-injection, DARPP32 and ubiquitin immunoreactivity were used to quantify the lesion volume and the number of ubiquitin-positive aggregates after sh39p10 or sh39p16 co-injection with htt171-82-exon39. The results are expressed as means ± SEM (n = 8). One-way ANOVAs were conducted for each SNP (**P<0.01; ***P<0.001). Newman-Keuls post-hoc comparison between control and matched shRNA groups concerning the size of the lesion showed significant efficacy for the shSNP only in position 16 (B). Regarding the number of aggregates (C), the four constructs were effective. However, for the mismatched conditions, the post-hoc test also revealed significant differences for both stainings revealing that these shSNP are not selective. (TIF) [file pone.0099341.s003.tif]
