# Supplementary material for: Allele-Specific Silencing of Mutant Huntingtin in Rodent Brain and Human Stem Cells
Source: PLoS One. 2014 Jun 13;9(6):e99341. doi: 10.1371/journal.pone.0099341 (PMC4057216; doi:10.1371/journal.pone.0099341)
Supplement: Table S1 — Sequences of the primers used to generate fragments of HTT gene containing each SNP. The sequences targeted by the shSNPs are in bold. The position of the SNP is in red. (DOC) [file pone.0099341.s004.doc]

Table S1.

| **Name** | **Sequence** |
| --- | --- |
| Exon39A-F | CTCGAGAGAGATG**GGGACAGTAATTCAACGCT**AG |
| Exon39C-F | CTCGAGAGAGATG**GGGACAGTACTTCAACGCT**AG |
| Exon39-R | CTCGAGCATATGGGCGTAATCTGGAACATCGTATGGGTATTCCTGGCAATAGAAAGTATGTTGCTGCTCACT |
| Exon50C-F | CTCGAGTGCT**CCCTCATCCACTGTGTGCA**C |
| Exon50T-F | CTCGAGTGCT**CCCTCATCTACTGTGTGCA**C |
| Exon50-R | CTCGAGCATATGGGCGTAATCTGGAACATCGTATGGGTAGCTGATGGCTTTTGGGGTATTTGTCCTTCTTTC |
| Exon60A-F | CTCGAGAACCA**GTTTGAGCTAATGTATGTG**A |
| Exon60G-F | CTCGAGAACCA**GTTTGAGCTGATGTATGTG**A |
| Exon60-R | CTCGAGCATATGGGCGTAATCTGGAACATCGTATGGGTAGCTGGGCAGGTGGCTGCTCCTGAGCGTGCTCTC |
| Exon67C-F | CTCGAGAGCCTTT**GGAAGTCTGCGCCCTTGTG**CC |
| Exon67T-F | CTCGAGAGCCTTT**GGAAGTCTGTGCCCTTGTG**CC |
| Exon67-R | CTCGAGCATATGGGCGTAATCTGGAACATCGTATGGGTACTCAGCCCCACCAGGACTGCAGACACTCCC |
