# Supplementary material for: Allele-Specific Silencing of Mutant Huntingtin in Rodent Brain and Human Stem Cells
Source: PLoS One. 2014 Jun 13;9(6):e99341. doi: 10.1371/journal.pone.0099341 (PMC4057216; doi:10.1371/journal.pone.0099341)
Supplement: Table S2 — Sequences of the oligonucleotides used to generate the shRNA targeting the SNP and the controls. The sense and anti-sense strands of the shRNA are given in bold. The position of the SNP is in red. (DOC) [file pone.0099341.s005.doc]

Table S2.

| **Name** | **Sequence** |
| --- | --- |
| shGFP | CTAGTTTCCAAAAA**AAGCTGACCCTGAAGTTCA**TCTCTTGAA**TGAACTTCAGGTCAGCTT**GGGGATCTGTGGTCTCATACAGAAC |
| shLuc | CTAGTTTCCAAAAA**CGTACGCGGAATACTTCGA**TGACAGGAAG**TCGAAGTATTCCGCGTACG**GGGGATCTGTGGTCTCATACAGAAC |
| shUNIV | CTAGTTTCCAAAAA**GTATCGATCACGAGACTAG**TGACAGGAAG**CTAGTCTCGTGATCGATAC**GGGGATCTGTGGTCTCATACAGAAC |
| sh39Ap10 | CTAGTTTCCAAAAA**GGGACAGTAATTCAACGCT**TGACAGGAAG**AGCGTTGAATTACTGTCCC**GGGGATCTGTGGTCTCATACAGAAC |
| sh39Ap16 | CTAGTTTCCAAAAA**GTAATTCAACGCTAGAA**GATGACAGGAAG**TCTTCTAGCGTTGAATTAC**GGGGATCTGTGGTCTCATACAGAAC |
| sh39Cp16 | CTAGTTTCCAAAAA**GTACTTCAACGCTAGAAGA**TGACAGGAAG**TCTTCTAGCGTTGAAGTAC**GGGGATCTGTGGTCTCATACAGAAC |
| sh39Cp10 | CTAGTTTCCAAAAA**GGGACAGTACTTCAACGCT**TGACAGGAAG**AGCGTTGAAGTACTGTCCC**GGGGATCTGTGGTCTCATACAGAAC |
| sh50C | CTAGTTTCCAAAAA**CCCTCATCCACTGTGTGCA**TGACAGGAAG**TGCACACAGTGGATGAGGG**GGGGATCTGTGGTCTCATACAGAAC |
| sh50T | CTAGTTTCCAAAAA**CCCTCATCTACTGTGTGCA**TGACAGGAAG**TGCACACAGTAGATGAGGG**GGGGATCTGTGGTCTCATACAGAAC |
| shHtt6 | CTAGTTTCCAAAAA**AGCTTTGATGGATTCTAAT**TCTCTTGAA**ATTAGAATCCATCAAAGCT**GGGGATCTGTGGTCTCATACAGAAC |
| sh60A | CTAGTTTCCAAAAA**GTTTGAGCTAATGTATGTG**TGACAGGAAG**CACATACATTAGCTCAAAC**GGGGATCTGTGGTCTCATACAGAAC |
| sh60G | CTAGTTTCCAAAAA**GTTTGAGCTGATGTATGTG**TGACAGGAAG**CACATACATCAGCTCAAAC**GGGGATCTGTGGTCTCATACAGAAC |
| sh67C | CTAGTTTCCAAAAA**GGAAGTCTGCGCCCTTGTG**TGACAGGAAG**CACAAGGGCGCAGACTTCC**GGGGATCTGTGGTCTCATACAGAAC |
| sh67T | CTAGTTTCCAAAAA**GGAAGTCTGTGCCCTTGTG**TGACAGGAAG**CACAAGGGCACAGACTTCC**GGGGATCTGTGGTCTCATACAGAAC |
