# Supplementary material for: Allele-Specific Silencing of Mutant Huntingtin in Rodent Brain and Human Stem Cells
Source: PLoS One. 2014 Jun 13;9(6):e99341. doi: 10.1371/journal.pone.0099341 (PMC4057216; doi:10.1371/journal.pone.0099341)
Supplement: Table S3 — Sequences of the primers used for RT-qPCR. (DOC) [file pone.0099341.s006.doc]

Table S3.

| **Name** | **Sequence** |
| --- | --- |
| hHtt-F | CTGCACCGACCAAAGAAAGAAC |
| hHtt-R | CATAGCGATGCCCAGAAGTTTC |
| hHtt-2F | ATGGACGGCCGCTCAGGTTCT |
| hHtt-2R | GCTCAGCACCGGGGCAATGAA |
| GFP-F | GACGTAAACGGCCACAAGTT |
| GFP-R | AAGTCGTGCTGCTTCATGTG |
| PPIA-F | ATGGCAAATGCTGGACCAAA |
| PPIA-R | GCCTTCTTTCACCTTCCCAAA |
| BACTIN-F | TGAAGGTGACAGCAGTCGGTTG |
| BACTIN-R | GGCTTTTAGGATGGCAAGGGAC |
| NeuN-F | CAACTCCACCCTTCCGACCC |
| NeuN-R | TCCCGAATTGCCCGAACAT |
| Aldh-F | TTCGCTGGCTGGTGTGATAAGA |
| Aldh-R | GGTCAAGGTCAGGTTGCGGTT |
