# Supplementary material for: Allele-Specific Silencing of Mutant Huntingtin in Rodent Brain and Human Stem Cells
Source: PLoS One. 2014 Jun 13;9(6):e99341. doi: 10.1371/journal.pone.0099341 (PMC4057216; doi:10.1371/journal.pone.0099341)
Supplement: Table S4 — List of primer sequences used for SNP sequencing in NSC. (DOC) [file pone.0099341.s007.doc]

**Table S4.**

| SNP sequencing | locus/primer name | forward primer sequence | reverse primer sequence | amplicon  size |
| --- | --- | --- | --- | --- |
|  | *HTT* exon 39 cDNA | TAGCCAAACAGCAGATGCAC | GTTCAAGCTGTCCAGGGTGT | 605 bp |
|  | *HTT* exon 39 gDNA | TCCGTGAGCACTGTTCAACT | TGAGGACAAGAGACAGAACATGA | 451 bp |
|  | *HTT* exon 50 cDNA | TCATTTGCACCTTCCTCCTGA | TTCTTCTGGTGGGCTCTCCTC | 763 bp |
|  | *HTT* exon 50 gDNA | CCTGTTGGCCATCTCTCACC | CCCCAAACGAAGGTACACGA | 959 bp |
|  | *HTT* exon 60 cDNA | CACCGAGCGCAACCAGTTT | TCCCAAGGACGGCAGCT | 131 bp |
|  | *HTT* exon 60 gDNA | GGCTTCAGCACAGAAACCTC | GGATTCTAACAGCGCGATTC | 248 bp |
| CAG | *HTT* Exon 1 | AAGGCCTTCGAGTCCCTCAAGTC | CGGCTGAGGCAGCAGCGGCT | 177 pb |
|  | *HTT* CAG | ATGAAGGCCTTCGAGTCCCTCAAGTCCTTC | GGCGGTGGCGGCTGTTGCTGCTGCTGCTGC | 110 pb |
| allele specific RT | locus/primer name | SNP | sequence | |
|  | *HTT* 39A | rs363125 | CTTCTAGCGTTCAAT | |
|  | *HTT* 39C |  | TTCTAGCGTTCAAG | |
|  | *HTT* 50C | rs362331 | GAAGTGCACACAGTG | |
|  | *HTT* 50T |  | GAAGTGCACACAGTA | |
|  | *HTT* 60A | rs2276881 | AGCGTCACATACATT | |
|  | *HTT* 60G |  | AGCGTCACATACATC | |
