# Supplementary material for: Allele-Specific Silencing of Mutant Huntingtin in Rodent Brain and Human Stem Cells
Source: PLoS One. 2014 Jun 13;9(6):e99341. doi: 10.1371/journal.pone.0099341 (PMC4057216; doi:10.1371/journal.pone.0099341)
Supplement: Materials and Methods S1 — (DOCX) [file pone.0099341.s008.docx]

**MATERIALS AND METHODS S1**

**Plasmids**

The fragments of HTT containing the SNP, used to generate the chimeric mutant HTT, were obtained by PCR using forward primers containing the sequence of HTT corresponding to the shSNP + 4 to 7 bp from HTT+ a XhoI restriction site; reverse primers consisting of 33bp of the following exon within HTT sequence + an HA tag + a XhoI restriction site (Table S1). The PCR fragments were ligated into pCR2.1-TOPO, and then extracted by XhoI digestion. The resulting XhoI fragments were inserted into the XhoI site downstream from htt171-82Q in the self-inactivating (SIN) transfer vector described previously [[1](#_ENREF_1)].

Oligonucleotides corresponding to the various shRNA and containing the sense-strand, a loop, the anti-sense strand, the stop codon as well as 17 nucleotides from the H1 promoter were synthesized (Table S2). The SNP was placed at the tenth position for rs363125, rs2276881 and rs362307 (sh39A, sh39C; sh60A, sh60G and sh67C, sh67T), eleventh position for rs362331 (sh50C, sh50T) and sixteenth position also for rs363125 (sh39Ap16, sh39Cp16)**.**

As controls, shRNA targeting EGFP (shGFP) or Luciferase (shLuc) or (shUNIV, no homologous sequence in the rodent genome) or the “AllStars Negative Control siRNA” from Qiagen (Transfection of NSC) were used. These oligos and the primer H1-3F: CACCGAACGCTGACGTCATCAACCCG were used for PCR with pBC-H1 (pBC plasmid; Stratagene, Amsterdam, The Netherlands) containing the H1 promoter as previously described [[2](#_ENREF_2)]. The PCR product was ligated into in pENTR/D-TOPO (Invitrogen, CergyPontoise, France). The LR clonase recombination system was used to transfer the H1-shRNA cassette to SIN-cPPT-PGK-GFP-WPRE-LTR-TRE-gateway (SIN-CWP-GFP-TRE-gateway) [[2](#_ENREF_2)].

**HEK 293T cell culture, transfection and western blot analysis**

HEK 293T cells were cultured in DMEM supplemented with 10% fetal bovine serum (FBS), 100 U/ml penicillin, and 100 µg/ml streptomycin at 37°C in 5% CO_2_/air atmosphere. One day prior to transfection, 293T cells were plated at a density of 750,000 cells per well in 6-well plates (Becton Dickinson, Le Pont De Claix, France). The cells were co-transfected by the calcium phosphate method with the SIN-W-PGK-htt171-82Q-exon (2.5µg) and shRNA targeting the matched SNP (efficacy) or mismatched SNP (selectivity). Controls included co-transfection with shGFP or shUNIV plasmids (shCtrl: 2.5 µg)(Table S2).

Western blot analysis: 48h after transfection, the 293T cells were harvested and lysed in 20mM Tris pH7.5, 2mM EDTA, 150mM NaCl, 1% NP40, with a protease inhibitor cocktail (Sigma-Aldrich,St. Louis, Missouri, United States). Protein concentration was determined by the Bradford method (Bio-Rad protein assay). Forty micrograms of the protein were run on 10% SDS-polyacrylamide gels, and transferred to Protran nitrocellulose membranes in 0.025M Tris, 0.192M glycine, and 20% ethanol. The membranes were blocked in TBST pH8.4 with 5% nonfat milk for 30min at room temperature (RT), and then incubated for 2 hrs with a 1:1000 dilution of the mouse anti-HA-11 antibody (MMS-101R, Covance, Rueil-Malmaison France). These blots were washed for 30min in TBST and incubated with an ECL anti-mouse-HRP antibody (1:5000; GE Healthcare Europe GmbH, Freiburg, Germany) for 30min at RT. After a 30min wash, bound antibody was detected using ECL Western Blotting Detection Reagents (GE Healthcare Europe GmbH, Freiburg, Germany). The blots were then washed and incubated for 15min at RT in 1x antibody stripping solution (Re-Blot: Western blot recycling kit, Chemicon International Inc., Temecula, USA). The blots were blocked a second time and incubated for 2h at RT with a 1:5000 dilution of the mouse anti-α-Tubulin antibody (T5168, Sigma, St. Louis, Missouri, USA). Secondary antibody and revelation steps were identical to those for HA-11 antibody.

To demonstrate the correlation between HTT mRNA and protein levels, new samples (n=3) were generated by transient transfection of 293T cells. The samples were collected at 48hrs and analyzed by Western blot (Odyssey Infrared Imaging, LI-COR) with an anti-HA-11 mouse monoclonal antibody (HA11 clone 16B12, 1:1000, Covance), anti-α-Tubulin rabbit polyclonal antibody (AB4074, 1:1000, Abcam) and the corresponding secondary antibodies (IRDye 680CW donkey anti-mouse, IRDye 800CW donkey anti-rabbit, 1:7000, LI-COR). RT-qPCR was performed as previously described. The control groups were used to determine the percentage of *HTT* mRNA and protein present in the samples and measure Bravais-Pearson correlation coefficients (Statistica, Statsoft, Maisons-Alfort, France).

**Stereotaxic injections of lentiviral vectors**

Concentrated viral stocks were thawed on ice and resuspended by repeated pipetting. The animals were anesthetized using a ketamine/xylazine solution (for rats: 75mg/kg ketamine + 10mg/kg xylazine, i.p, and for mice: 15mg/kg ketamine + 1.5mg/kg xylazine, i.p). Lentiviral vectors were stereotaxically injected into the striatum of animals through a 34-gauge blunt-tip needle linked to a Hamilton syringe by a polyethylene catheter. The stereotaxic coordinates for an intrastriatal injection for rats/mice were [[3](#_ENREF_3),[4](#_ENREF_4)]: 0.5/0.5mm rostral to bregma, 3/2mm lateral to midline and 4.5/3.5mm from the duramater/skull surface [[3](#_ENREF_3),[4](#_ENREF_4)]. The viral particle contents were matched to 1,000ng p24 antigen for single infections with chimeric *HTT*; 300ng (rats) or 250ng (mice) p24 antigen for single infection of shRNA. For co-infections (ratio 1:1), 600ng p24 antigen of total lentivirus was injected. Régulier et al. 2002, showed that co-injection of two lentiviruses resulted in a high proportion of striatal neurons expressing both transgene [[5](#_ENREF_5)].

NSCs genotyping and linkage of SNP and the CAG expansion

*Single Nucleotide Polymorphism (SNP) mapping.*Genomic DNA prepared using Promega’s Wizard genomic purification Kit and cDNAs were used. The regions flanking SNPs rs363125 (exon 39), rs362331 (exon 50) and rs2276881 (exon 60) were amplified using platinum Pfx polymerase and primers flanking the SNPs (Table S4). The PCR products were then sequenced by Beckman Coulter Genomics (Takeley, United Kingdom) to analyze SNP status at rs363125, rs362331, and rs2276881.

*SNP allele-specific reverse transcription and CAG amplification.*Allele specific primers for *HTT* or Random primers (2pmol) and the SuperScript III reverse transcription kit (Invitrogen, CergyPontoise, France) were used for reverse transcription of *HTT* mRNA. We modified the manufacturer’s protocol: RNA-primer mix incubation at 65°C for 15 min, elongation at 55°C for 90 min. Primers were designed with SNP site at the 3’ end with Vector NTi (Table S4). Following reverse transcription, the resulting allele-specific cDNAs were diluted (1/10, 1/100) and used as templates for CAG tract amplification. A section of exon 1 with CAG repeats was amplified twice using *HTT* Exon1 and *HTT* CAG primers and Takara LA-Taq DNA Polymerase with GC Buffer II (Takara Bio, Madison, Wisconsin, USA) according to the manufacturer’s instructions, with a modified PCR program: an initial denaturation step (98°C, 3min) followed by 32 amplification cycles (98°C, 30 sec; 55°C, 30 sec; 72°C, 2 min) and 72°C, 5 min.

*CAG repeat size analysis.* To analyze CAG repeat sizes and to test for associations with SNP heterozygosity for each sample, we ran PCR products on an Agilent 2100 Bioanalyser with the Agilent DNA 1000 Kit (Agilent technologies, Massy, France). The expected sizes of the amplicons for a normal allele carrying 23 CAG repeats is 108 bp for *HTT* exon1 primers and 41 bp for *HTT* CAG primers. The number of CAG repeats was therefore calculated using the equations: *X=(Y-108)/3* for *HTT* exon1 primers, and equation: *X=(Y-41)/3* for *HTT* CAG primers (X being the number of CAG repeats and Y being the size of the amplicon determined by analysis on the Agilent chip).

Small doses of anti-specific cDNAs were produced during allele specific RT-PCR, especially from the mutated allele because the enzymatic reaction is less efficient. Therefore, when analyzing allele-specific PCR amplicon from mutated alleles, an additional peak corresponding to the normal allele is likely to be detected by the Agilent chip. The converse should not be true.

*siRNA nucleofection*. Small interfering RNAs (siRNAs) were used to nucleofect NSCs with the “Rat Neural Stem Cell Nucleofector Kit” (VPG-1005 Kit Amaxa/Lonza, Levallois, France) according to the manufacturer's protocol. Briefly, 5 million NSCs re-suspended in 100 μL of nucleofection solution with 1.5 μg (1µM) of siRNA, were electroporated with an AmaxaNucleofector (Amaxa/Lonza, Levallois, France) and then plated onto Polyornithine/Laminin-coated dishes. The NSCs were incubated at 37°C, 5% CO_2_ for 24 hours before RNA extraction and RT-qPCR [[6](#_ENREF_6)]. Each experiment was performed in quadruplicate, for both cell lines (Huez2.3 and SIVF018). Primer sequences for *HTT* were 5’-AGTGATTGTTGCTATGGAGCGG and 5’-GCTGCTGGTTGGACAGAAACTC.

**Videomicroscopy**

Stacks of 4-6 images with a Z-step of 0.3 µm were acquired with a 100 X PlanApo N.A. 1.4 oil immersion objective coupled to a piezo device (PI). Images were collected every 1s in stream mode using a Micromax camera (Ropper Scientific) set at 2 X 2 binning with an exposure time of 100ms. All stacks were treated by automatic batch deconvolution using the PSF of the optical system. ImageJ software (<http://rsb.info.nih.gov/ij/>, NIH, USA) was used for projections, animations and analyses. Kymographs were generated with ImageJ and a homemade plug-in. Linear trajectories were selected and drawn on kymographs and recorded in the ROI manager. A dedicated homemade plug-in was used for automatic analyses of trajectories (F.P. Cordelières, IC).

Immunofluorescence

After videomicroscopy, the neuronal cultures were washed with PBS and fixed for 20 minutes at 37°C with PFA 4%. The cultures were then incubated in a blocking solution supplemented with 1% BSA and 0.03% Triton X-100 (Sigma, St. Louis, Missouri, USA), and again at 4°C overnight in blocking solution containing the primary monoclonal antibody anti-HTT-2166 (clone 1HU-4C8) (MAB2166, 1:200, Chemicon International Inc., Temecula, USA). They were washed in PBS, and incubated at room temperature with AlexaFluor-647 secondary antibody (Molecular Probes, Invitrogen, CergyPontoise, France).

**Statistical analyses**

Data were analyzed using GraphPad Prism 4 and Statview 4.5 (SAS Institute Inc.). One-way analysis of variance (ANOVA) followed by appropriate post hoc test was used to determine statistically significant differences (set at p < 0.05). Data are presented as means ± SEM.

**SUPPLEMENTARY REFERENCES**

1. de Almeida LP, Ross CA, Zala D, Aebischer P, Deglon N (2002) Lentiviral-mediated delivery of mutant huntingtin in the striatum of rats induces a selective neuropathology modulated by polyglutamine repeat size, huntingtin expression levels, and protein length. J Neurosci 22: 3473-3483.

2. Drouet V, Perrin V, Hassig R, Dufour N, Auregan G, et al. (2009) Sustained effects of nonallele-specific Huntingtin silencing. Ann Neurol 65: 276-285.

3. Paxinos G, Watson C (2004) The Rat Brain in Stereotaxic Coordinates; Edition F, editor. New York: Academic press. 1-474 p.

4. Franklin B, Paxinos G (1996) The Mouse Brain in Stereotaxic Coordinates; Press A, editor. New York. 1-350 p.

5. Régulier E, Pereira de Almeida L, Sommer B, Aebischer P, Déglon N (2002) Dose-dependent neuroprotective effect of CNTF delivered via tetracycline-regulated lentiviral vectors in the quinolinic acid rat model of Huntington's disease. Hum Gene Ther 13: 1981-1990.

6. Feyeux M, Bourgois-Rocha F, Redfern A, Giles P, Lefort N, et al. (2012) Early transcriptional changes linked to naturally occurring Huntington's disease mutations in neural derivatives of human embryonic stem cells. Hum Mol Genet 21: 3883-3895.
